# Experimental study on corrosion resistance of coiled tubing welds in high temperature and pressure environment

**DOI:** 10.1371/journal.pone.0244237

**Published:** 2021-01-22

**Authors:** Shaohu Liu, Liu Yuanliang, Zhong Hong, Zou Jiayan, Yang Dong

**Affiliations:** 1 School of Mechanical Engineering, Yangtze University, Jingzhou, PR, China; 2 State Key Laboratory of Oil and Gas Reservoir Geology and Exploitation, Southwest Petroleum University, Chengdu, PR, China; China University of Mining and Technology, CHINA

## Abstract

Coiled tubing (CT) has been widely used for oil and gas exploitation, however corrosion of CT under high pressure and high temperature (HPHT) environment was often reported, also corrosion induced failures of CT welds were often observed to occur during service. Corrosion related behaviors of CT welds are not clear. Therefore, a study of the corrosion resistance of CT welds under HPHT environment is carried out. In order to efficiently evaluate the corrosion resistance of welds, some test samples were obtained by linear cutting out of a CT110 in service on the site. The water samples from gas field were used as the test reagent to simulate the actual corrosive medium. Based on the results of weight loss test under HPHT corrosive environment and tensile test under room conditions, the corrosion sensitivities of the welding seam and base material under various temperatures and partial pressures of CO_2_ as well as the mechanical properties of the corroded CT were compared and evaluated quantitatively, the corrosion morphologies and material products of the test samples were analyzed by scanning electron microscope (SEM). The test results showed that the corrosion rates of the welding seam in a HPHT caldron were 1.7, 2.0 and 1.2 times of the base metal’s when the total pressure is 4MPa, and the temperature is 30°C, 60°C and 90°C, respectively. The corrosion rates of the welding seam is 2.0, 2.1 and 2.0 times of the base metal’s when the partial pressure of CO_2_ is 0.1MPa, 0.2MPa and 0.3MPa, respectively. The yield strength of the weld seam after corrosion test was found to be reduced by 4.8% (the yield strength of the base metal was reduced by 4.0%) and its tensile strength was reduced by 8.2% (the base metal was reduced by 7.1%). This indicates that CT weld seam is more susceptible to corrosion than CT base material under service condition.

## Introduction

In order to better explore offshore oil and gas resources, CT has been widely used in the fields of well cleaning, drilling, workover, completion, mechanical production, well logging perforation and oil and gas transportation in recent years. In the past 20 years, CT technology has attracted plenty of attention of the offshore oil and gas industry and has been widely recognized. International oil companies such as BJ, Baker Hughes, Halliburton, Schlumberger and Shell have deployed many CT offshore operations in the Norwegian North Sea, the Gulf of Mexico, the West Indian Sea, the Brunei Sea, the Arabian Sea, the Brazilian Sea and the South China Sea [1,2].

CT is often welded with low carbon alloy steel bars, also known as flexible tubing. A reel of CT can reach a length of thousands of meters [3,4]. With the increase of drilling depth, the service environment of CT is becoming worse and worse, and it is gradually developing towards the trend of larger diameter, higher strength and corrosion resistance [5].

There are various forms of CT failures caused by hostile service environment conditions [6–9]. Crabtree and Service Company have made statistical analysis on the failure causes of CT in recent 20 years, and found that the main failure forms were corrosion, mechanical damage and fatigue damage [10,11], especially the corrosion induced failure forms. According to the statistical results by Crabtree from 1997 to 2007, the corrosion induced CT failures were reported as 33% of the total failures, and the work from service companies from 2007 to 2017 showed the proportion of corrosion induced failures over total failures is arround 34% [12]. Therefore, it is important and necessary to further study the corrosion mechanism of the CT.

Scholars have conducted a lot of research on the corrosion failure mechanism of CT materials, especially of the base metal. Zhu Chenglong et al. [13] simulated the corrosion mechanism of QT-900 CT base metal in CO_2_ environment and evaluated the effect of temperature on its corrosion related behaviors. Liu Ming and Xue Yuna et al. [14,15] studied the electrochemical corrosion behavior of CT in highly mineralized aqueous environment by means of potentiodynamic polarization and impedance spectroscopy, and quantitied the effect of immersion time on corrosion rate. Van arnam et al. [16–18] studied the corrosion resistance of CT90 and CT100 by using linear polarization resistance method and tafel curve generation method. Bi Zongyue et al. [19] analyzed the fracture failure of CT80 through physical and chemical performance inspection, microstructure analysis and energy spectrum analysis, and found mainly due to the combined effect of tensile stress, periodic plastic strain and acidic corrosion environment in the well. Luo Sheji et al. [20] analyzed the electrochemical corrosion behavior of CT80 base material and weld NaCl solution with different mass fraction through electrochemical corrosion test. Liu Shaohu et al. [21] studied the corrosion behavior of coiled tubing weld seam and base metal at different temperatures through electrochemical corrosion experiment and finite element analysis method, and then studied the influence of area ratio of weld and base metal, weld residual height and defect on corrosion. However, most of the ongoing research of CT corrosion are focused on the corrosion of the raw materials of CT processing, while the corrosion research of CT110 welds in actual service conditions after forming are less reported.

**Fig 1:**
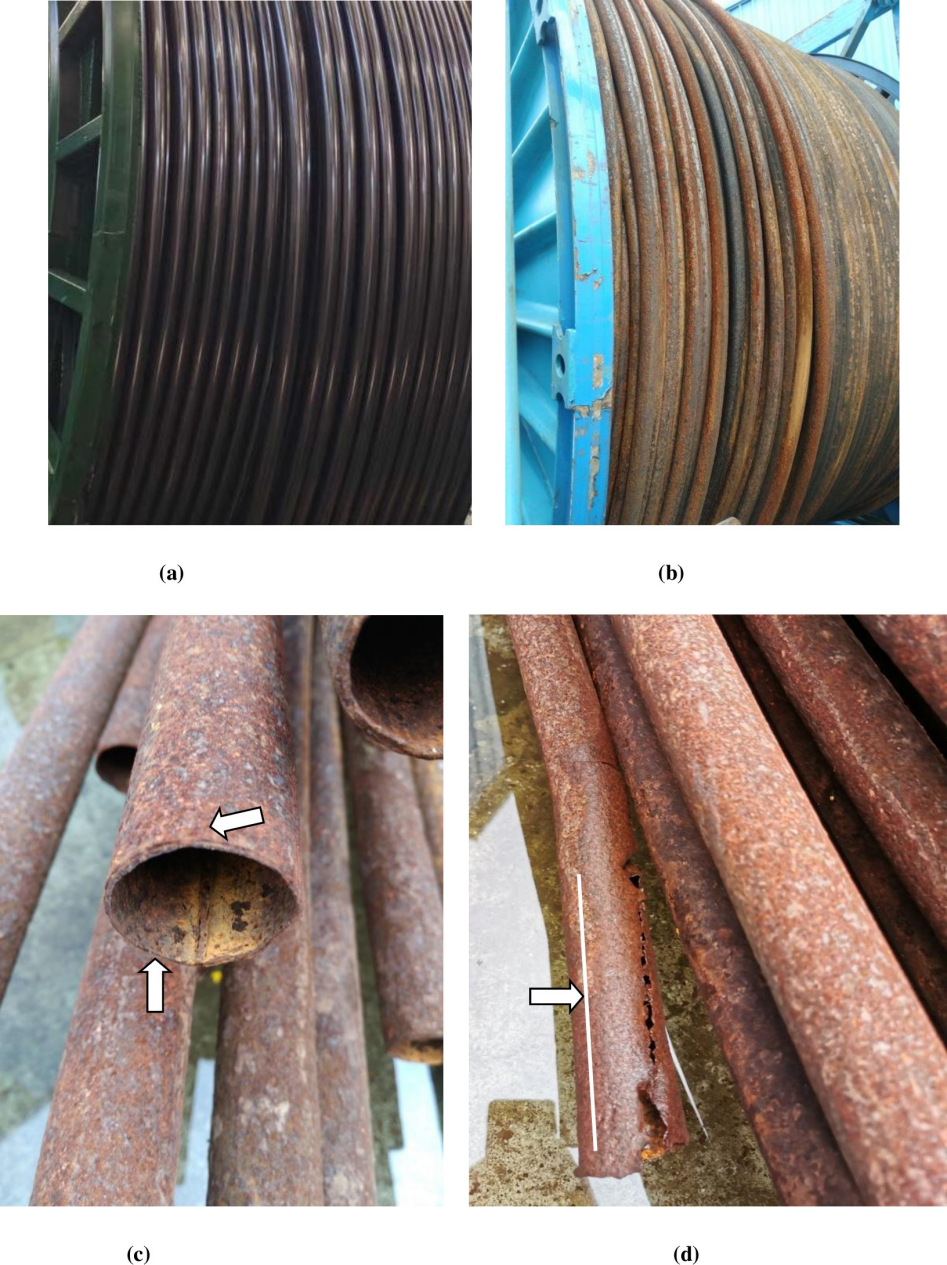
Corrosion morphology of CT after operation in Marine environment. (a) A new CT (not used), (b) After use CT, (c) Appearance of CT after corrosion, (d) Corrosion cracking along the weld.

In addition to the harsh operating environment in offshore oil fields, CT corrosion problems during storage and transportation under high salt and moisture environment are more prominent, which severely affects the service life of CT and even causes more complex downhole accidents and safety related issues during operation. Fig 1 shows the corrosion morphology of CT after one offshore operation. Fig 1(A) shows the new CT, never been deployed in field. Fig 1(B) shows the picture of CT after one well operation in the offshore environment. The outer surface of the integral CT is maroon, and the overall corrosion development of the CT is very severe. Fig 1(C) shows the interior and exterior wall surfaces of the CT after corrosion. Many pits are visible on the surface of the CT. Fig 1(D) shows the appearance of corrosion cracking at the weld of CT. The crack is through the entire CT and is about 10mm away from the weld. It ends at the location where the corrosion pits developed. It can be seen from this that the welding seam of the CT is seriously corroded, which is likely to lead to fracture and other accidents. Therefore, it is very necessary to study the corrosion mechanism of the CT.

In this paper, the corrosion behavior of CT110 weld and base metal were studied by corrosion weight loss test in high-temperature and high-pressure boiler. An adaptability evaluation of corrosion severity of CT welds and base metal was conducted, also the impact of corrosion on the mechanical properties of CT was studies by tensile mechanical property test.

## Experiment

### Test sample

**Fig 2:**
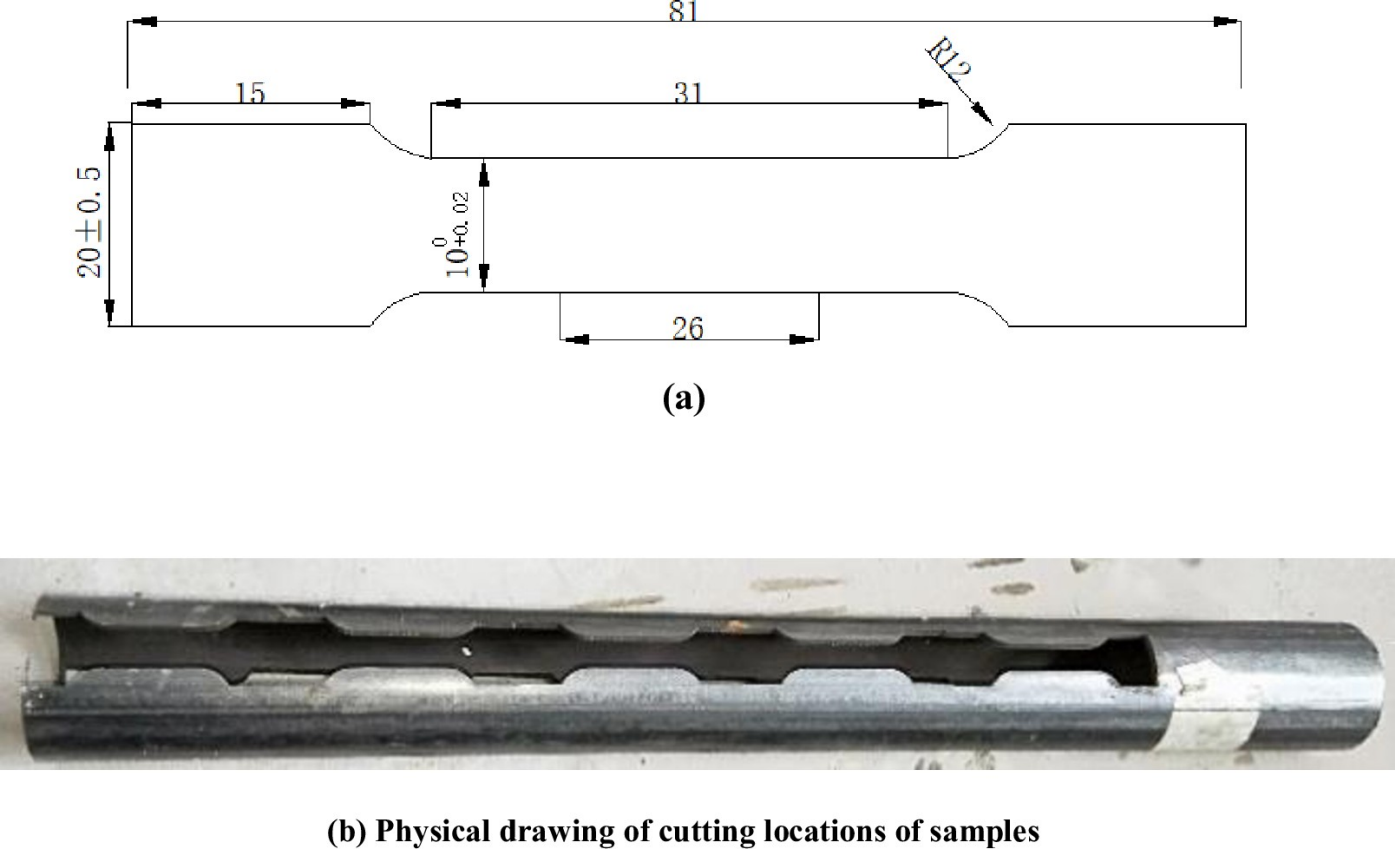
Sample figure. (a)Standard tensile specimen dimension and (b) Physcal drawing of cutting position of sample.

**Fig 3:**
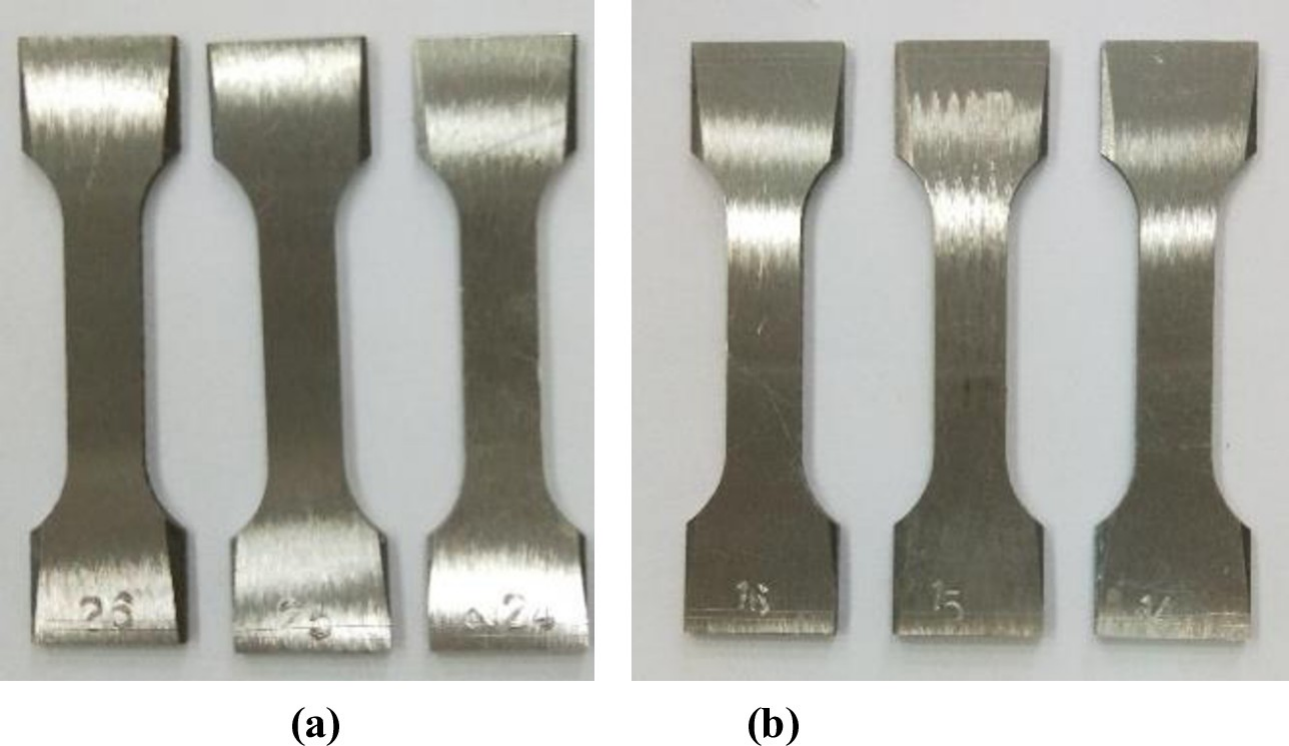
Standard tensile test samples of the WM and BM of CT. (a) WM, (b) BM.

Samples made of CT110 were used in the experiment, with an outer diameter of 50.8mm and a wall thickness of 4.4mm. The samples are defined as weld metal samples (WM) and base metal samples (BM), with their main chemical compositions shown in Table 1. According to *GB/T228-2002* national standard for tensile test specimen, the standard specimen with the dimension of 81mm×20mm×2mm [22] was processed by wire cutting out of WM and BM after forming, as shown in Fig 2(A). The WM samples were cut along the axis direction of the weld seam, and the CT weld line was arranged to align with the middle of the samples. The actual cutting locations of the sample were shown in Fig 2(B). The surfaces of the processed samples were polished and treated with 600^#^, 800^#^ and 1200^#^ water sandpaper successively, and final CT110 WM and BM samples were shown in Fig 3.

**Table 1:** CT110 WM and BM chemical composition (mass fraction %).

Element	C	Si	P	S	Cr	Mn	Fe
BM	0.14	0.39	0.12	0.05	0.56	0.48	allowance
WM	0.13	0.36	—	0.08	0.46	0.71	allowance

### Experimental reagents

The water sample was taken from Dabei gas field and was used as test reagent [23]. The test solution was made of three-stage distilled water. The drug content and ion concentrations of the test solution were shown in Table 2. After preparing the solution, add boric acid to adjust the solution pH to 5.6, and then keep the solution in a dry and cool place for standby.

**Table 2:** Ion concentration of experimental reagent and drug.

ion	Concentration(g/L)	drug	Weight (g)
*Ca*^2+^	7.03	*Cacl*_2_	19.5
*Mg*^2+^	0.76	*MgCl*_2_·6*H*_2_*O*	6.4
$SO_{4}^{2-}$	0.67	*Na*_2_*SO*_4_	1.0
$HCO_{3}^{-}$	0.90	*NaHCO*_3_	0.5
*Cl*^-^	60.0	*NaCl*	74.89

### Corrosion weight loss test

**Fig 4:**
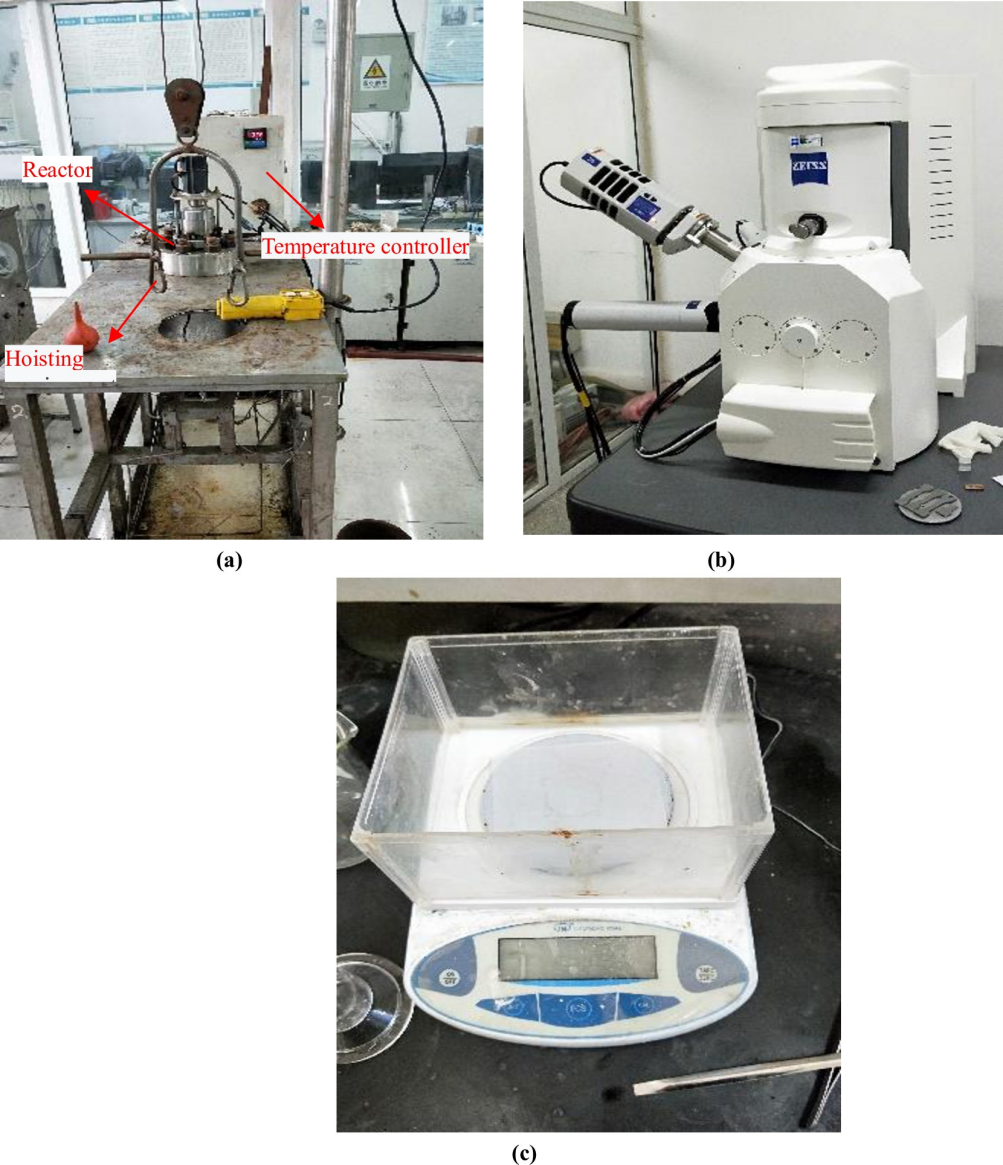
Main instruments of the experiment. (a) High-temperature and high-pressure reactor, (b) Scanning electron microscope and (c) Electronic balance.

During the experiment, the sample were hanged on the support with nylon wires and loaded into the high-temperature and high-pressure reactor (as shown in Fig 4(A), and the prepared experimental reagent was poured in, so that the sample in the reactor became fully soaked, then closed the reactor to start following test procedure: A pressure test was conducted in the reactor to ensure that all the seals were intact, and N_2_ was aerated to purge oxygen out of the reactor for 2 hours. Then adjusted the temperature to simulate the designed downhole conditions, injected CO_2_ to boost the pressure to the required level, and adjusted the total pressure to 4MPa. After all conditions were met started time recording and turned off the corrosion cycle in 96 hours. After releasing the reactor pressure and temperature to room conditons, took out the corroded sample, put it on the drying plate and wait to dry, visully checked the micro morphologies of the corrosion products on the surface of the CT samples through scanning electron microscope (as shown in Fig 4(B), and analyzed the element content with the matching EDS apparatus. Finally, put it in the plate to dry again, then weigh it on the electronic balance (as shown in Fig 4(C)).

According to *GB/10124-88* [24], the material corrosion rate is calculated by the weight loss method, with the following formula:

$$V_{corr}=\frac{87600\cdot \left(M_{2}‐M_{1}\right)}{\rho ST}$$ (1)

Where *V_corr_*, is the corrosion rate, mm/year; M_2_ is the sample mass before the test, g; M_1_ is the sample mass after the test, g; S is the total surface area of the sample, cm^2;^ T is the test time, s; ρ is the density of the material, g/cm^3^.

### Tensile mechanical properties test

**Fig 5:**
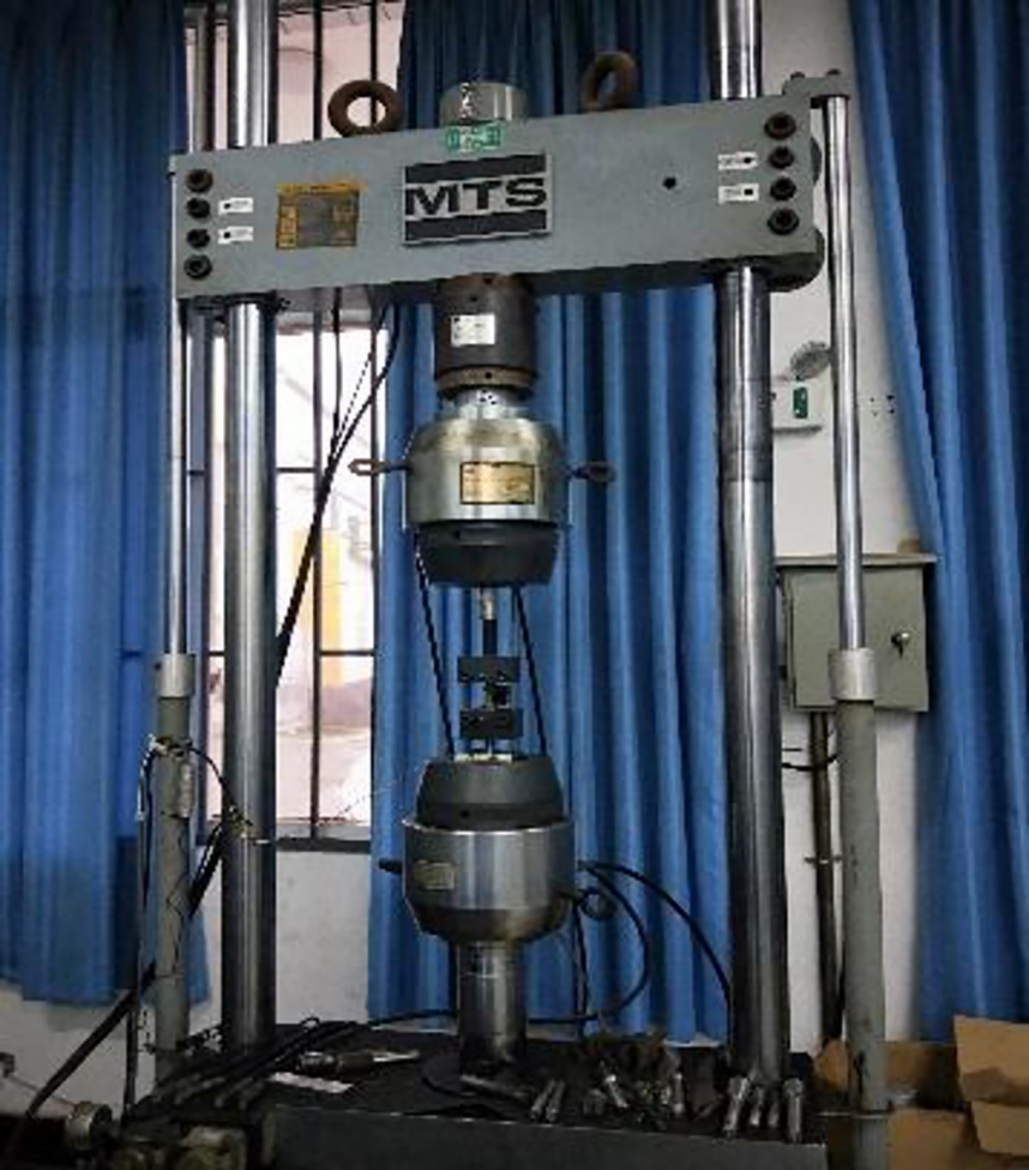
MTS hydraulic universal testing machine.

The MTS hydraulic universal testing machine (as shown in Fig 5) was used for tensile mechanical property test. According to API value rule [25], the stress-strain curve of the sample was drawn, and the yield strength, tensile strength and fracture elongation of the sample before and after corrosion were obtained. Then the mechanical property parameters of the WM and BM before and after corrosion were compared and analyzed against each. The yield strength was coresponding to 0.2% of the strain value, the tensile strength was the maximum stress before the plastic strain, and the fracture elongation was the strain value when the material broke.

## Result and discussion

### Corrosion morphology and products

**Fig 6:**
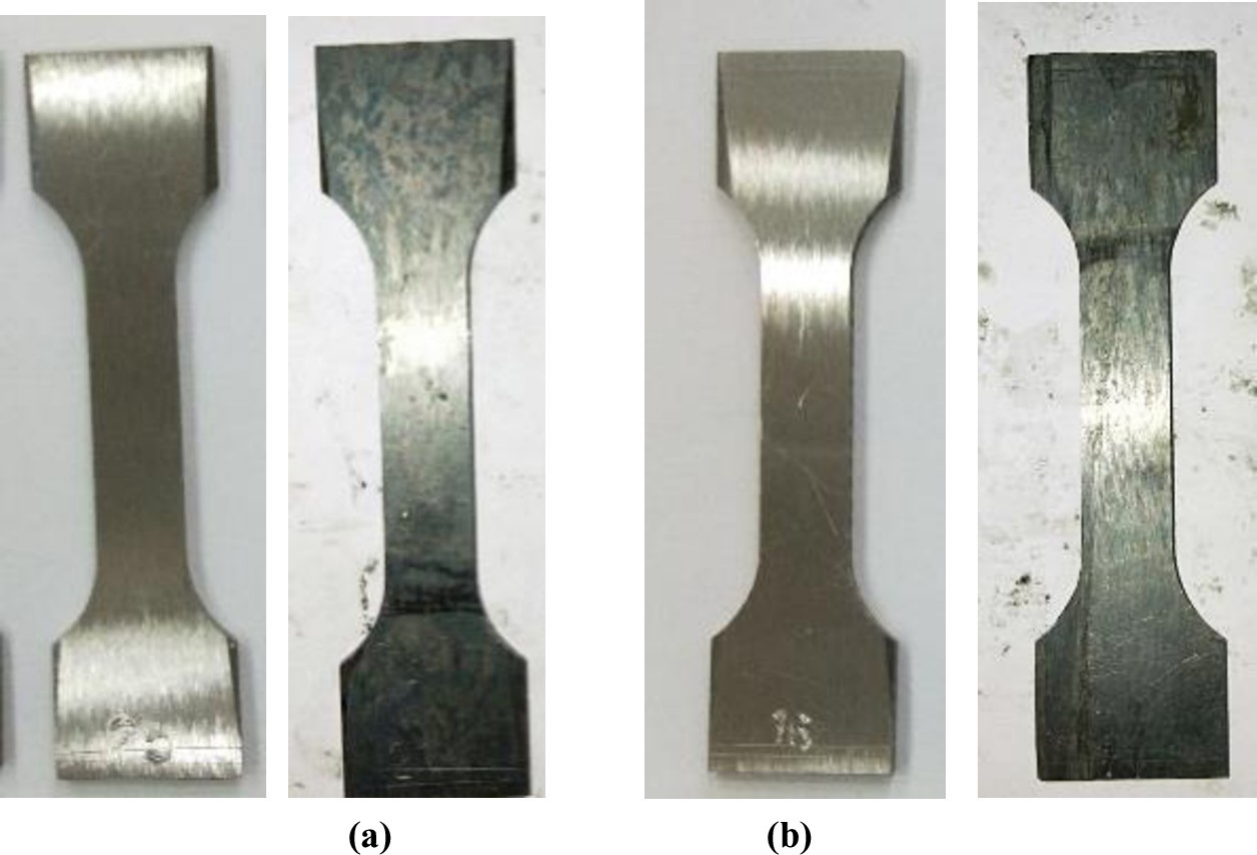
Comparison of macromorphology of sample surface before and after corrosion. (a)WM morphology and (b)BM morphology before and after corrosion.

Fig 6 was a comparison of macro morphology of WM and BM before and after corrosion. It can be seen from the figure that the surface of WM and BM sample were silvery white before corrosion, with bright color and smooth surface. After corrosion, the surface of the whole sample became gray and black, the metallic luster was dim, and some areas showed pitting. Through the comparison of the morphologies of WM and BM before and after corrosion, it can be seen that, the surface of BM was much smoother, and a lot of corrosion pits initiated on WM surface, which indicated that the corrosion of WM was more serious than that of BM.

**Fig 7:**
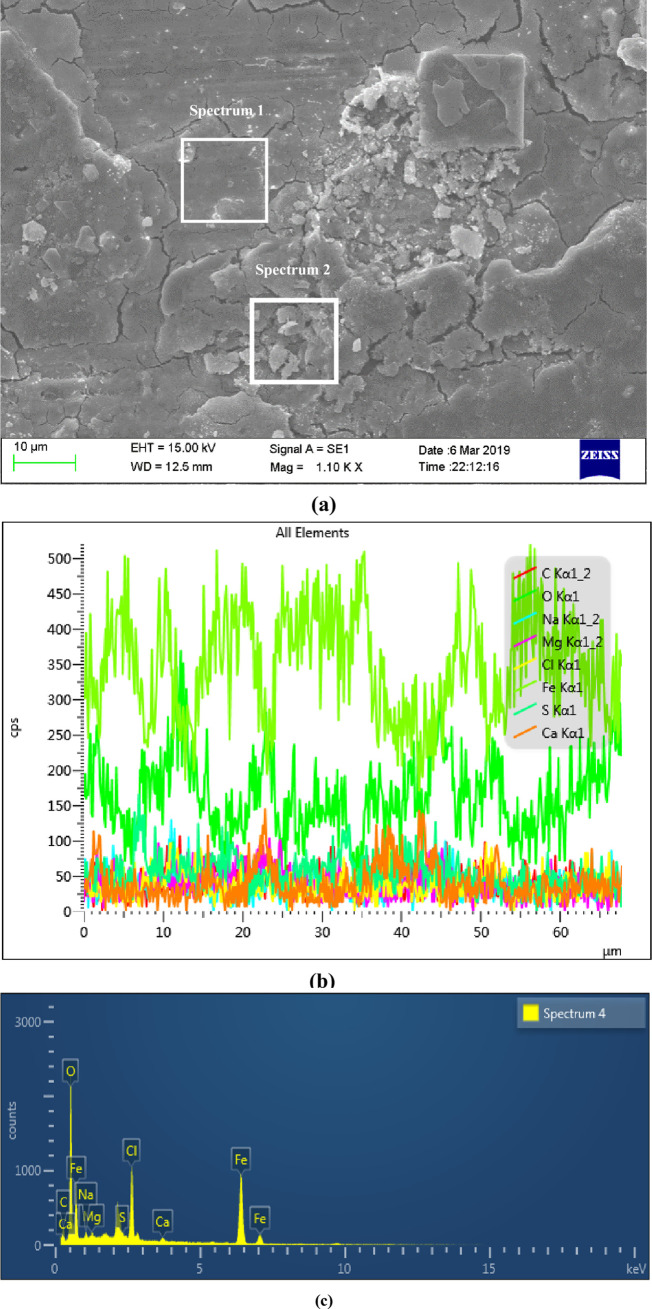
The micro morphology of sample surface and *EDS* region energy spectrum analysis diagram after BM corrosion. (a) Surface micro corrosion morphology, (b) Line scan of corrosion product elements on the surface and (c) Distribution of corrosion products on the surface.

**Fig 8:**
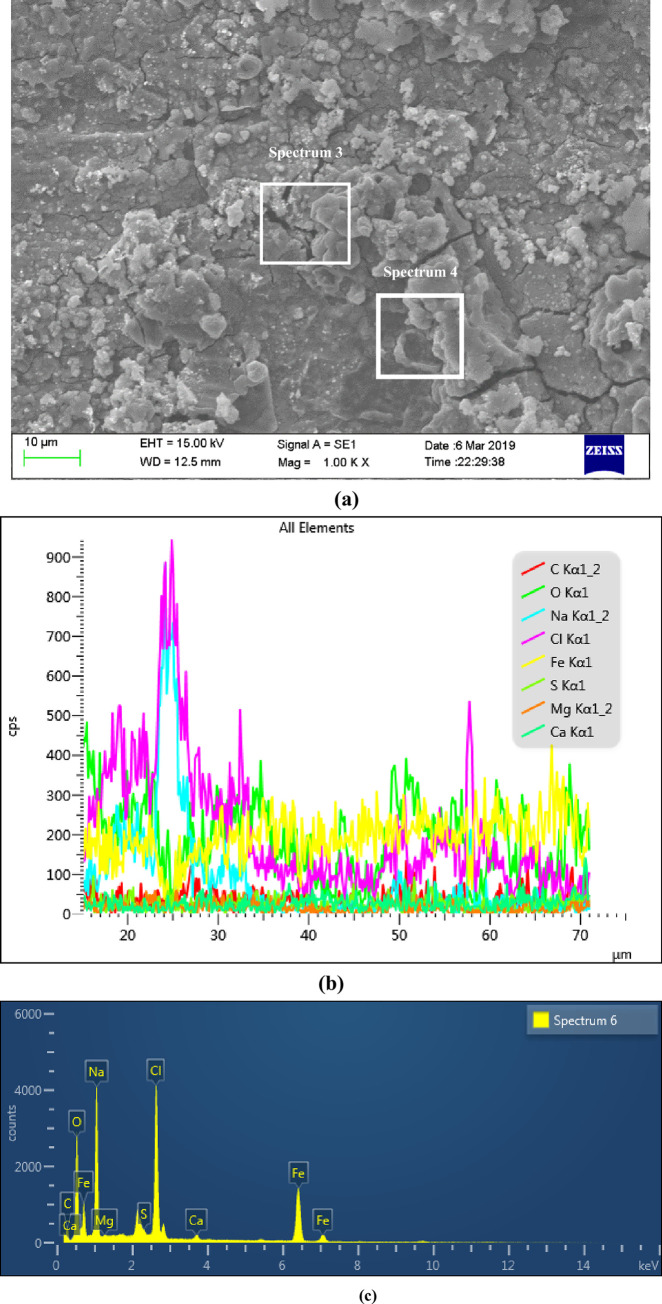
The micro morphology of sample surface and *EDS* region energy spectrum analysis diagram after WM corrosion. (a) Surface micro corrosion morphology, (b) Line scan of corrosion product elements on the surface and (c) Distribution of corrosion products on the surface.

Figs 7 and 8 were the surface micro morphologies and energy spectrum analysis diagram of BM and WM samples after corrosion, respectively. It can be seen from the micrograph after BM corrosion that there were less corrosion products on the surface of BM, and the corrosion product film in some surface areas had broken. It can be seen from the micrograph after WM corrosion that the WM surface were covered by a thicker layer of corrosion products, and most of the corrosion products film were broken, even falling off. It can be seen from the energy spectrum analysis diagram and element content table after the corrosion of BM and WM (as shown in Table 3), the compositions of the BM and WM corrosion products were similar, mainly composed of C, O, Fe, Na, Mg, Cl and Ca. according to the element content analysis of the surface, the content of C,O and Fe accounted for the most, and it can be concluded that the corrosion products were mainly C,O and Fe. While the other elements could possibly be the electrolytes which were sticked to the surface of the sample, instead of completely dissolved in the corrosion solution. Therefore, it can be determined that the corrosion products were the oxides of FeCO_3_ and iron. The formation of FeCO_3_ was due to that the CO_2_ gas dissolved in water to form carbonic acid, and then react with the Fe in the material to form FeCO_3_. While the oxides of iron might be formed by the oxidation of the corroded sample when exposed to air. Besides, the CL content level on WM surface was almost twice of that on BM. The main reason was that the WM surface was rough, with defects such as inclusions and stress concentration, which led to the fact that the passivation film of WM was not as stable as BM’s. The penetration ability of Cl^-^ in the corrosion solution was strong, and it was easier to be absorbed by WM surface, which undermined the stability of the corrosion product film, thus accelerating WM corrosion.

**Table 3:** The analysis results of surface energy spectrum of CT BM and WM after corrosion.

Element	C	O	Na	Mg	Cl	Ca	Fe	Total
BM	9.74	36.32	1.07	0.61	11.52	0.72	39.72	100
WM	9.34	22.35	20.78	0.15	20.75	0.84	25.79	100
Change/%	4.3	62.5	-94.9	75.4	-44.5	14.3	54	-

### Corrosion behavior of WM and BM at different temperature

**Fig 9:**
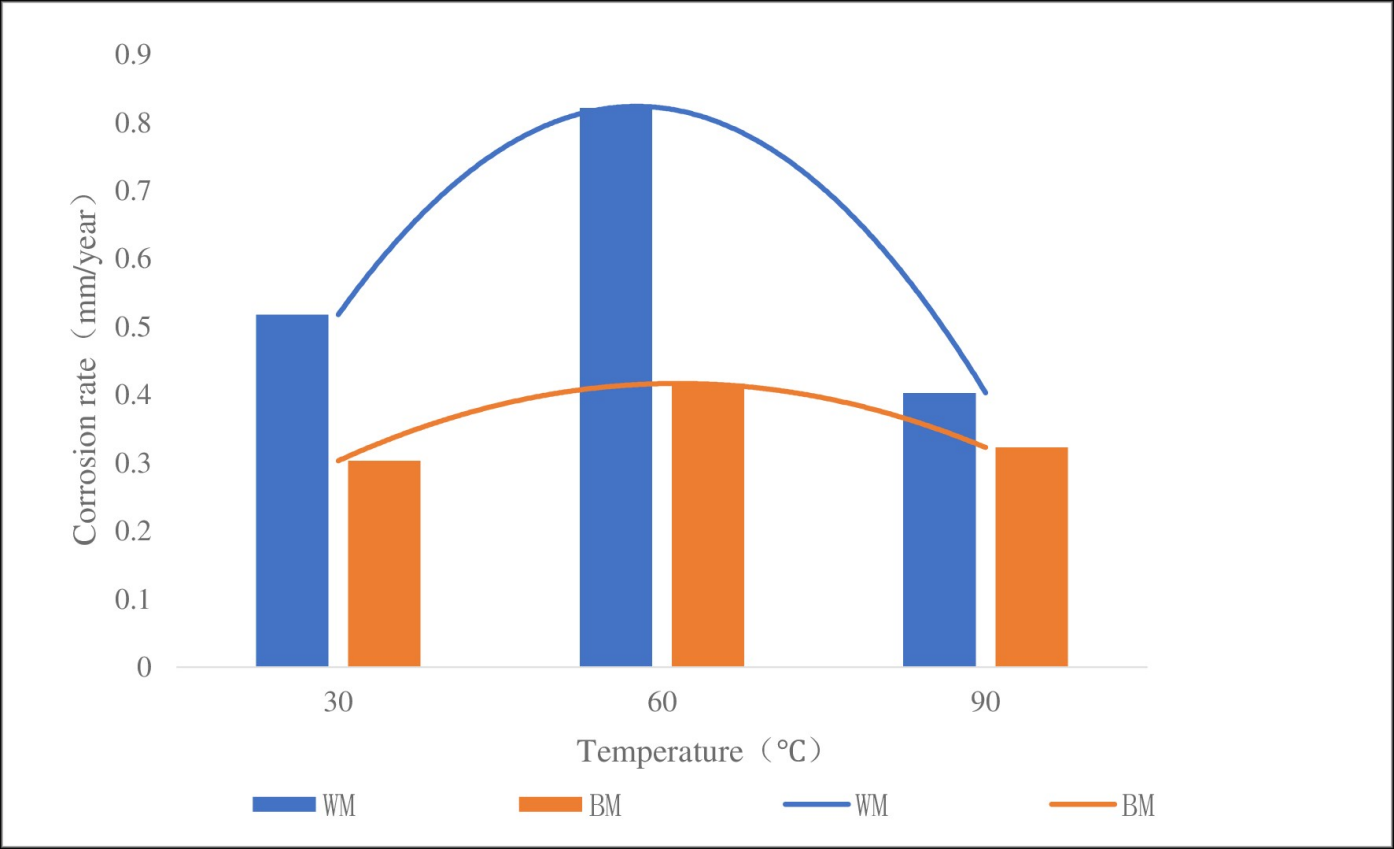
CT110 corrosion rate at different temperatures.

Fig 9 was a column chart of corrosion rate of the samples at different temperatures with CO_2_ partial pressure of 0.3 MPa, total pressure of 4 MPa, solution pH of 5.6. It can be seen from the figure that the corrosion rate of the weld at 30°C, 60°C and 90°C was 1.7, 2.0 and 1.2 times of that of the base metal, respectively. At 60°C, the corrosion rate was larger, showing a trend of increasing first and then decreasing. According to the principle of electrochemistry, the corrosion rate increases with the increase of temperature, when it reaches a certain peak value, the product formed by the corrosion reaction on the surface of the sample forms a dense protective film, thus hindering the occurrence of the corrosion reaction and reducing the corrosion rate. Through the comparison of the corrosion rate of base metal and weld metal, it can be seen that the corrosion rate of weld metal was higher than that of base metal at different temperature, which indicated that the weld was more susceptible to corrosion. The main reason was that during the welding process, the structure of the weld was not uniform, there was a certain residual stress after welding, and there were also some defects such as inclusions and dislocations [26], which caused the lattice distortion energy at the weld boundary, the activity to increase and the electrode potential to decrease, to create a potential difference between the weld and the base metal, and a galvanic cell will be formed this way to accelerate into a more severe corrosion on WM material.

### Corrosion behavior of WM and BM at different CO_2_ partial pressure

**Fig 10:**
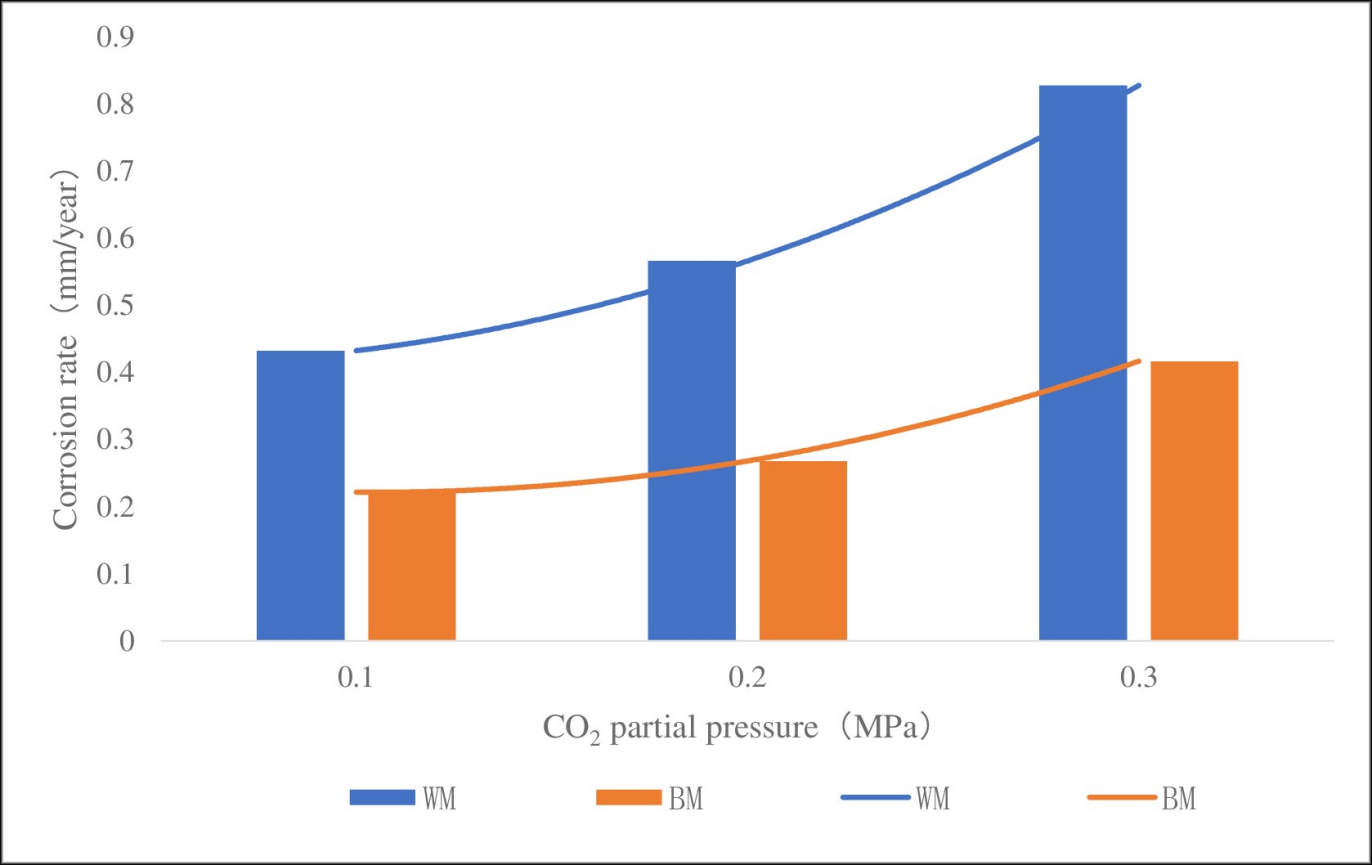
CT110 corrosion rate at different CO_2_ partial pressure.

Fig 10 was a column chart of corrosion rate of the sample at 60°C, 4 MPa, pH 5.6 and different CO_2_ partial pressure. It can be seen from the figure that the corrosion rate of WM at 0.1MPa, 0.2MPa and 0.3MPa was 2.0, 2.1 and 2.0 times of BM’s respectively, but the corrosion rate of WM and BM increased with the increase of CO_2_ partial pressure. It could be well explained from the principle of electrochemistry and the diffusion principle of substance. When the partial pressure of CO_2_ in the solution increases, the diffusion rate of CO_2_ molecules in the solution will increase accordingly to accelerate the reaction of the cathode, then both the concentration of carbonic acid generated and the concentration of H^+^ ionized will increase, which will further increase the dissolving activities of the metal iron at the anode and the general corrosion rate. It can also be concluded that the corrosion rate of WM is higher than that of BM at different CO_2_ partial pressure, which further proves that WM is more susceptible to corrosion.

### Analysis of tensile mechanical properties of samples

**Fig 11:**
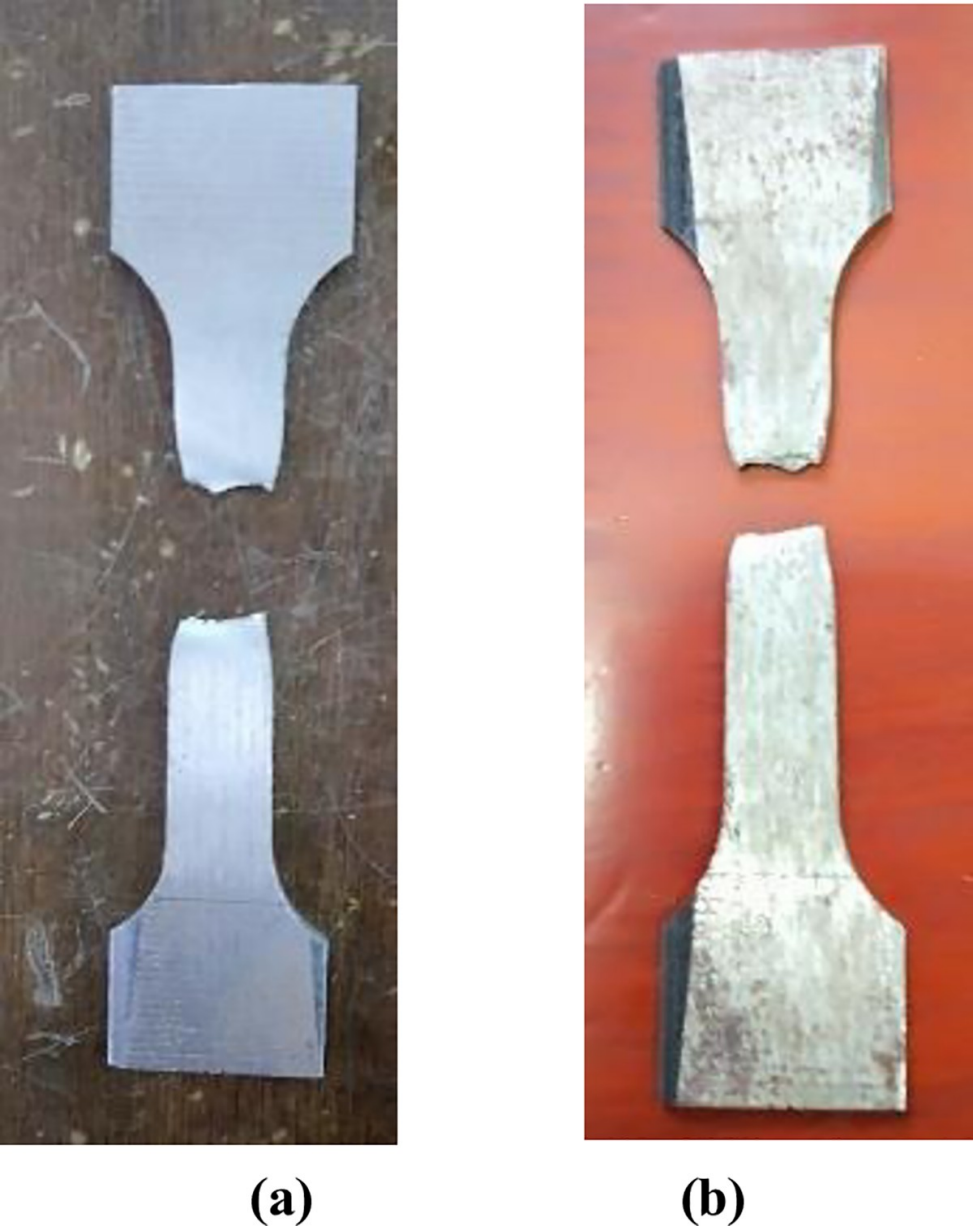
Fracture morphology of sample after tensile test. (a) Before corrosion and (b)After corrosion.

**Fig 12:**
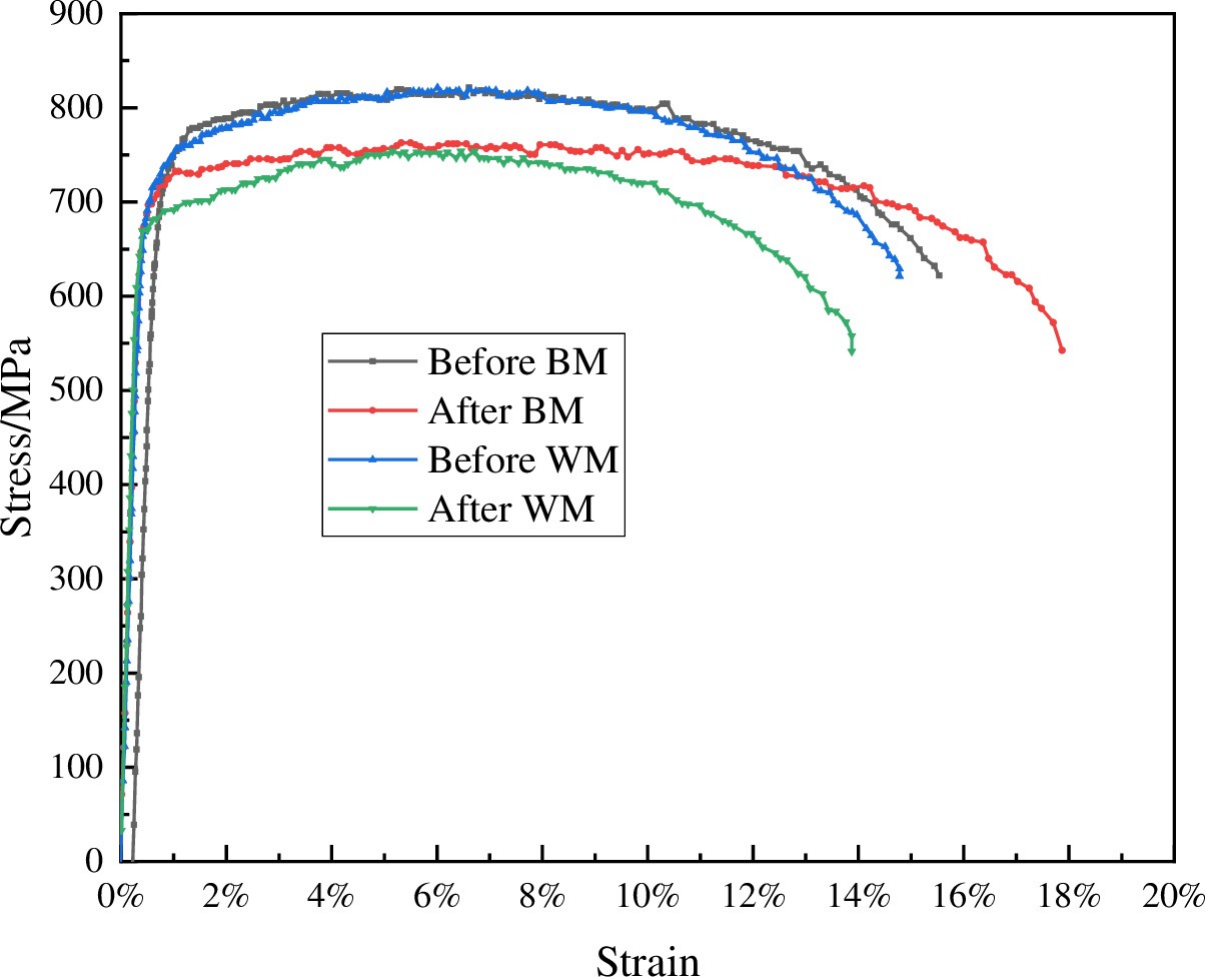
CT110 WM and BM stress-strain curve before and after corrosion.

It can be seen from Fig 11 that there were many dimples on the fracture surface of the sample before corrosion and the sample showed pretty good ductility. While the fracture surface of the sample after corrosion was relatively flat and tends to brittle fracture. Fig 12 showed the yield strength, tensile strength and elongation at break of the WM and BM before and after corrosion. Table 4 shows that the yield strength and tensile strength of CT110 weld were reduced by 4.8% and 8.2% respectively. The yield strength and tensile strength of CT110 base metal reduced by 4.0% and 7.1%, respectively. From the comparison of the above data, it can be concluded that corrosion undermined the mechanical properties of CT110, and showed a greater impact on the mechanical properties of the weld.

**Table 4:** The comparison of mechanical properties of CT110 WM and BM before and after corrosion.

sample	condition	yield strength (MPa)	tensile strength (MPa)	ratio	Elongation
WM	before	715.82	821.33	0.87	14.40%
	after	681.08	753.84	0.90	14.00%
BM	before	726.30	821.56	0.88	15.15%
	after	697.81	762.82	0.91	17.56%

## Conclusion

1. On the premise of meeting the standard, the WM and BM samples are made of the CT in service. This experimental study shows that the corrosion rate of the weld of high-strength CT110 is about twice of that of base metal, with a lower corrosion resistance than base’s.
2. At 30°C, 60°C and 90°C, the corrosion rate of the weld is 1.7, 2.0 and 1.2 times of that of the base metal, respectively. At 0.1MPa, 0.2MPa and 0.3MPa, the corrosion rate of the weld is 2.0, 2.1 and 2.0 times of that of the base metal, respectively.
3. After corrosion, the yield strength and tensile strength of the weld are reduced by 4.8% and 8.2%, the yield strength and tensile strength of the base metal are reduced by 4.0% and 7.1%, respectively. The mechanical strength of the weld decreased due to corrosion.
4. It is suggested to improve the mechanical properties of CT by improving the heat treatment method, or to suppress the corrosion activities by surface anti-corrosion treatment, and to maintain a more efficient monitoring and evaluation of the weld.

## Supporting information

S1 Fig Corrosion morphology of CT after operation in Marine environment. (a) A new CT (not used), (b) After use CT, (c) Appearance of CT after corrosion, (d) Corrosion cracking along the weld.
(TIF)

S2 Fig Sample figure. (a)Standard tensile specimen dimension and (b) Physcal drawing of cutting position of sample.
(TIF)

S3 Fig Standard tensile test samples of the WM and BM of CT. (a) WM, (b) BM.
(TIF)

S4 Fig Main instruments of the experiment. (a) High-temperature and high-pressure reactor, (b) Scanning electron microscope and (c) Electronic balance.
(TIF)

S5 Fig MTS hydraulic universal testing machine. (TIF)

S6 Fig Comparison of macromorphology of sample surface before and after corrosion. (a)WM morphology and (b)BM morphology before and after corrosion.
(TIF)

S7 Fig The micro morphology of sample surface and *EDS* region energy spectrum analysis diagram after BM corrosion. (a) Surface micro corrosion morphology, (b) Line scan of corrosion product elements on the surface and (c) Distribution of corrosion products on the surface.
(TIF)

S8 Fig The micro morphology of sample surface and *EDS* region energy spectrum analysis diagram after WM corrosion. (a) Surface micro corrosion morphology, (b) Line scan of corrosion product elements on the surface and (c) Distribution of corrosion products on the surface.
(TIF)

S9 Fig CT110 corrosion rate at different temperatures. (TIF)

S10 Fig CT110 corrosion rate at different CO_2_ partial pressure. (TIF)

S11 Fig Fracture morphology of sample after tensile test. (a) Before corrosion and (b) After corrosion.
(TIF)

S12 Fig CT110 WM and BM stress-strain curve before and after corrosion. (TIF)

S1 Table CT110 WM and BM chemical composition (mass fraction %). (DOCX)

S2 Table Ion concentration of experimental reagent and drug. (DOCX)

S3 Table The analysis results of surface energy spectrum of CT BM and WM after corrosion. (DOCX)

S4 Table The comparison of mechanical properties of CT110 WM and BM before and after corrosion. (DOCX)

S1 File (XLSX)

S2 File (XLSX)

S3 File (XLSX)
