# Supplementary figures and images for: Experimental study on corrosion resistance of coiled tubing welds in high temperature and pressure environment
Source: PLoS One. 2021 Jan 22;16(1):e0244237. doi: 10.1371/journal.pone.0244237 (PMC7822278; doi:10.1371/journal.pone.0244237)

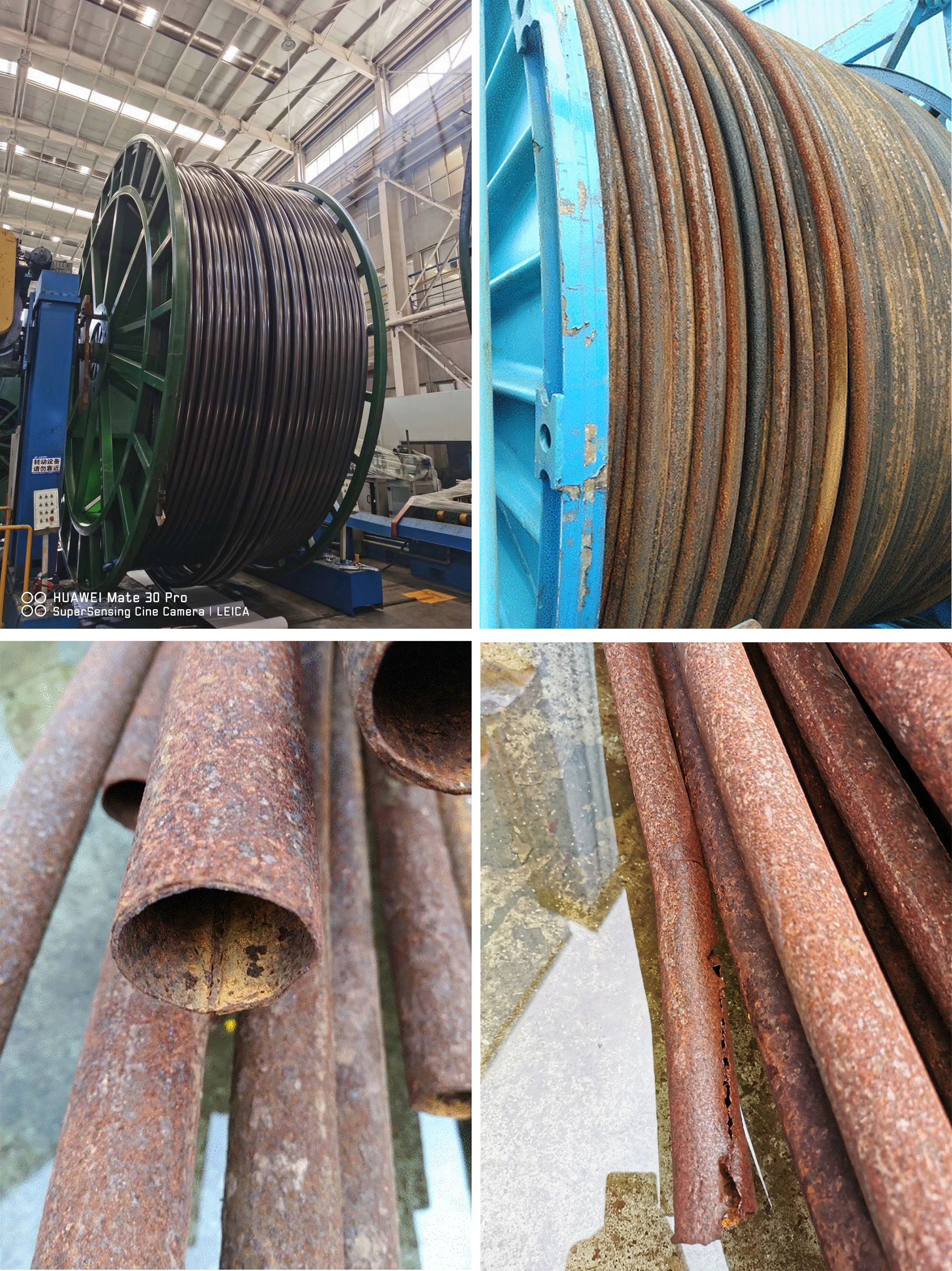

Supplement: S1 Fig — (a) A new CT (not used), (b) After use CT, (c) Appearance of CT after corrosion, (d) Corrosion cracking along the weld. (TIF) [file pone.0244237.s001.tif]

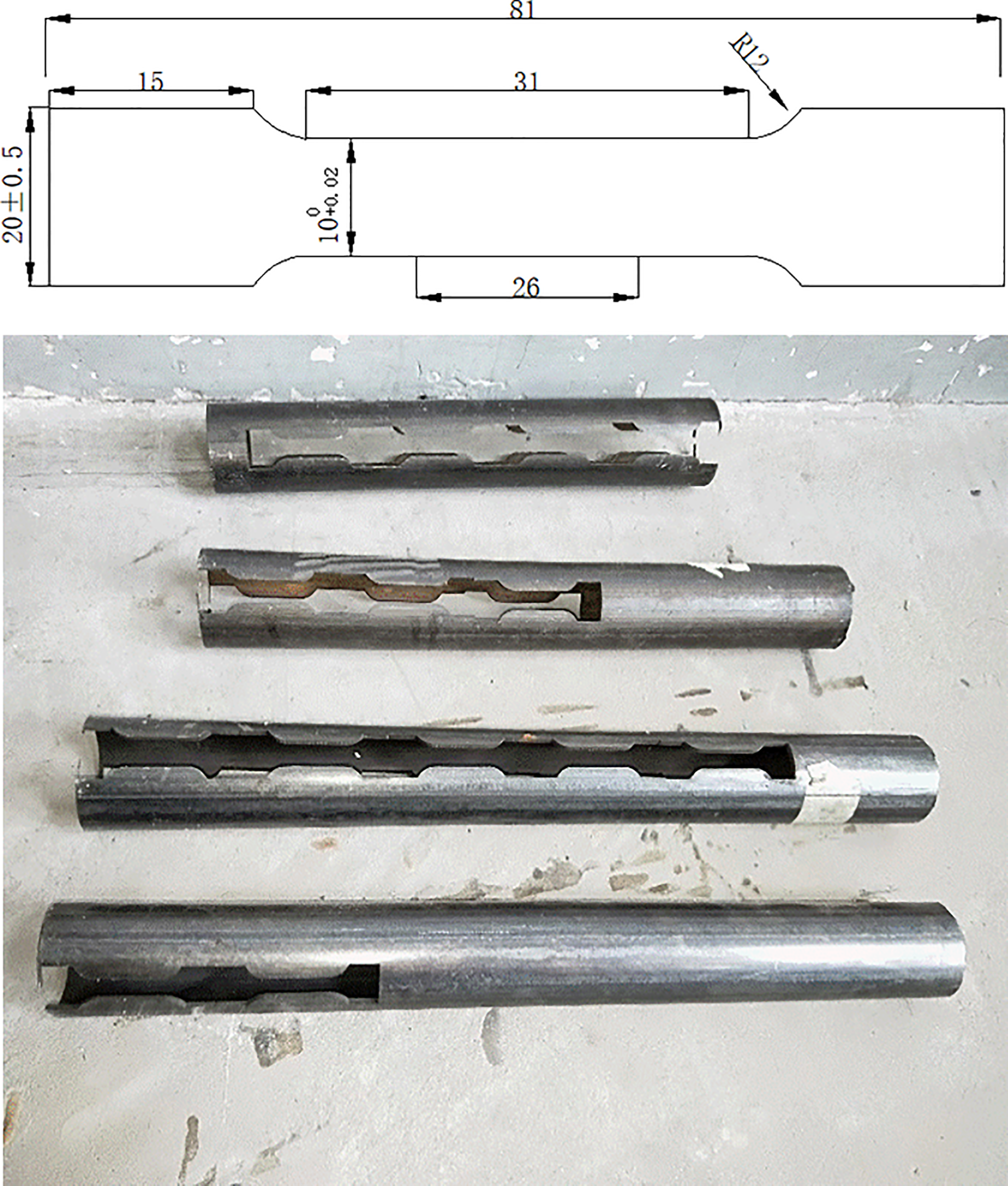

Supplement: S2 Fig — (a)Standard tensile specimen dimension and (b) Physcal drawing of cutting position of sample. (TIF) [file pone.0244237.s002.tif]

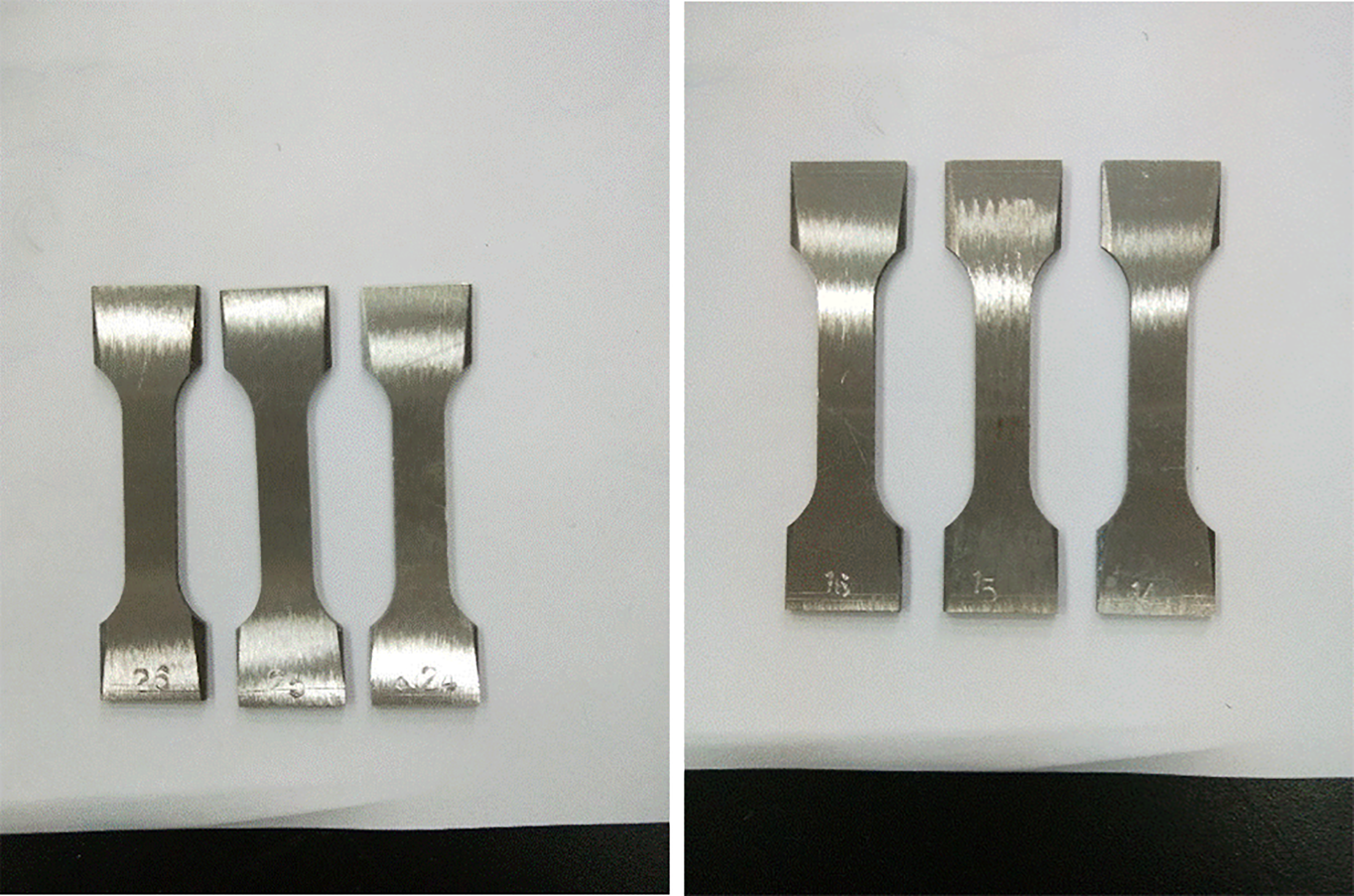

Supplement: S3 Fig — (a) WM, (b) BM. (TIF) [file pone.0244237.s003.tif]

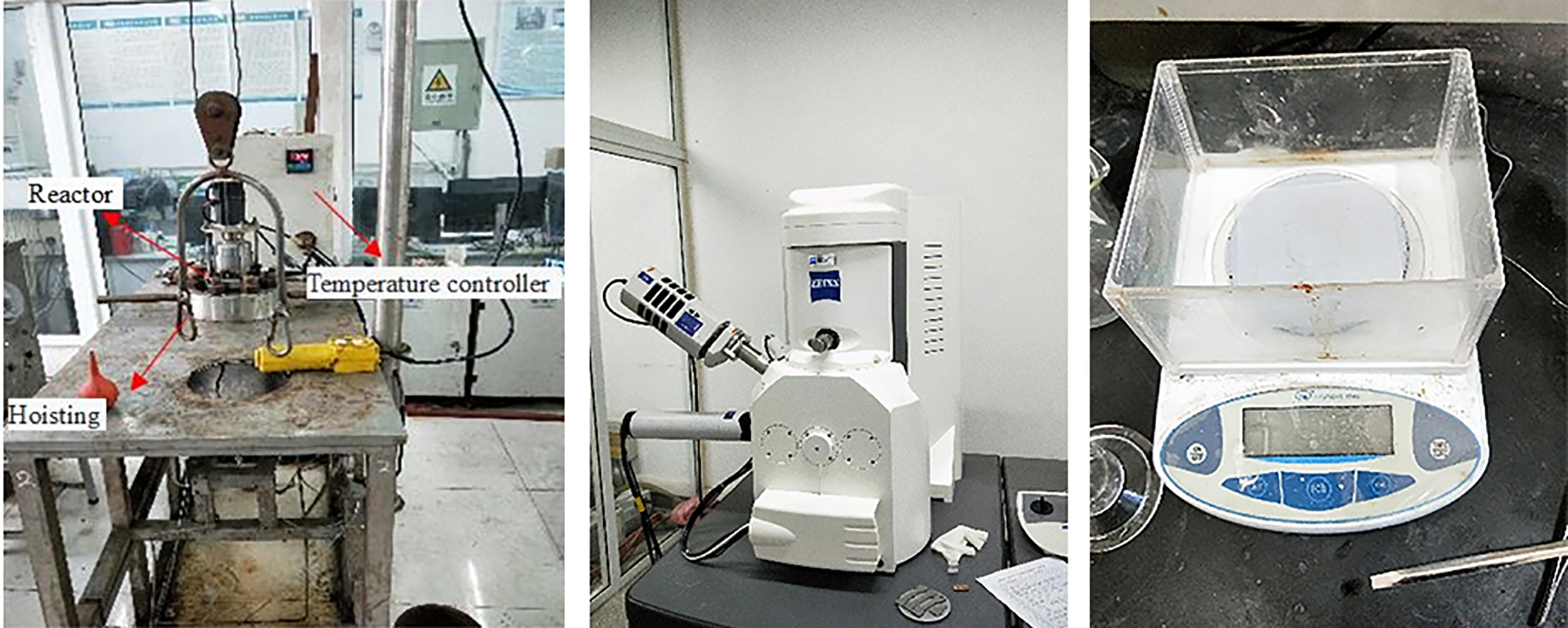

Supplement: S4 Fig — (a) High-temperature and high-pressure reactor, (b) Scanning electron microscope and (c) Electronic balance. (TIF) [file pone.0244237.s004.tif]

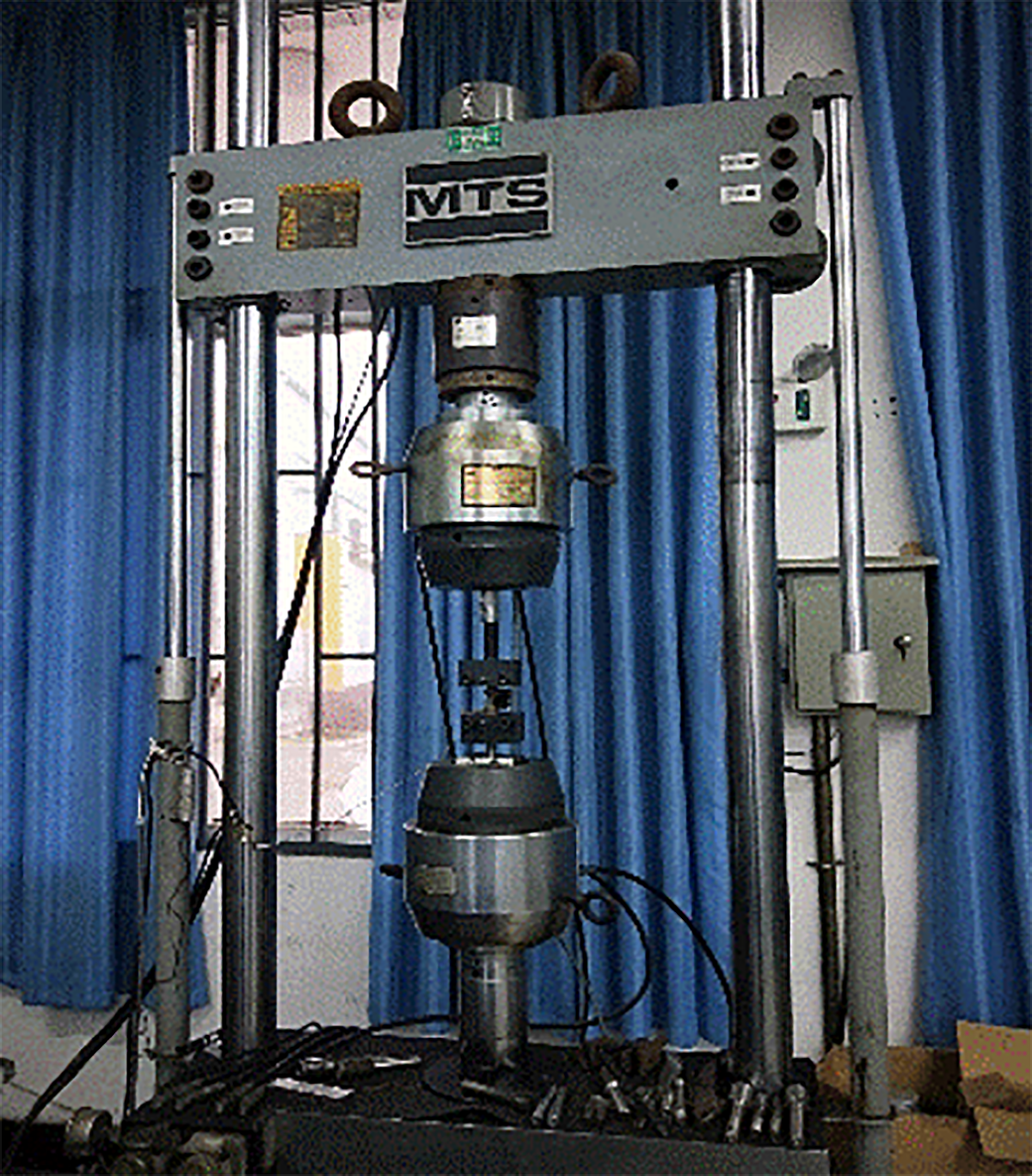

Supplement: S5 Fig — (TIF) [file pone.0244237.s005.tif]

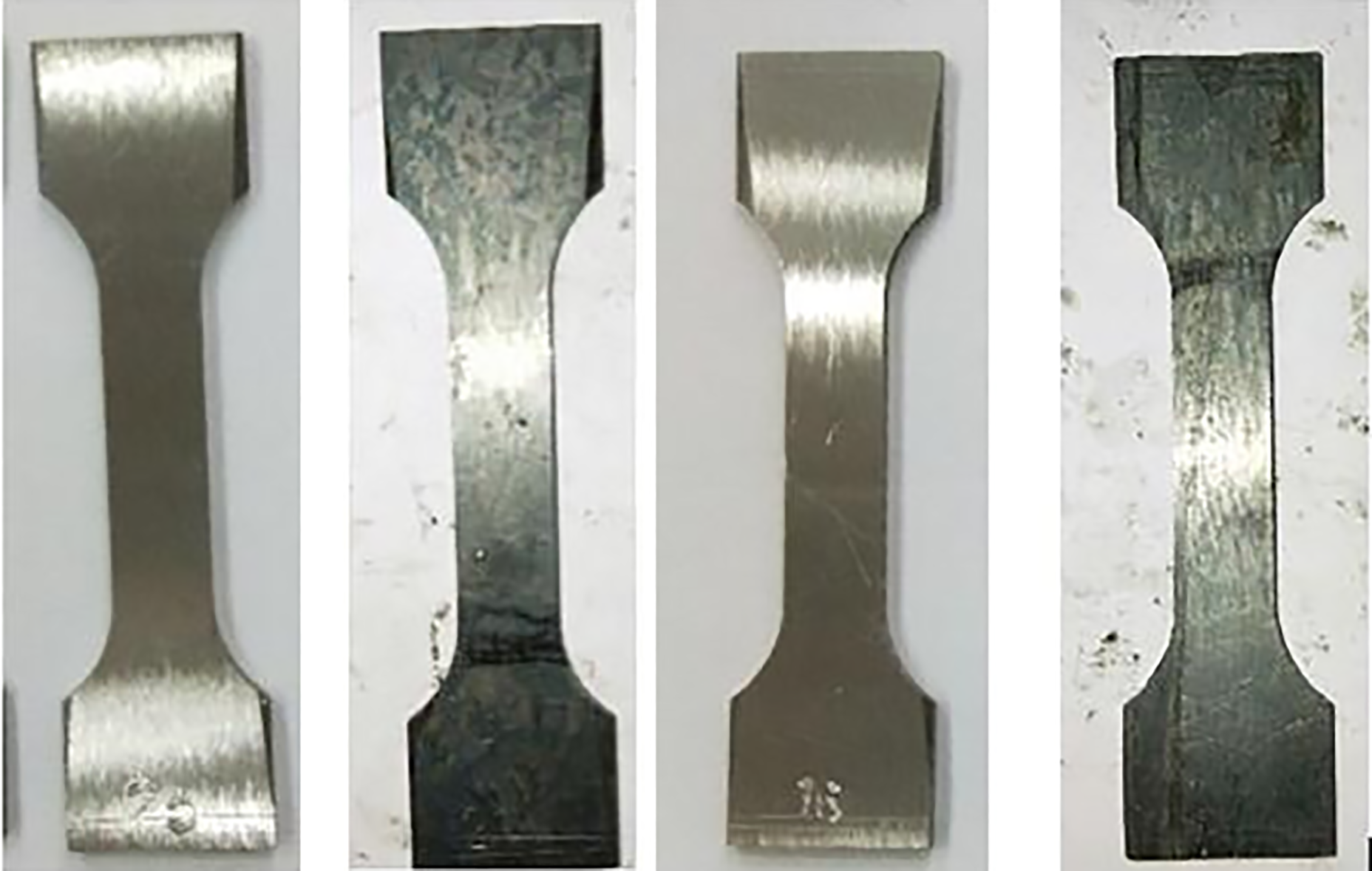

Supplement: S6 Fig — (a)WM morphology and (b)BM morphology before and after corrosion. (TIF) [file pone.0244237.s006.tif]

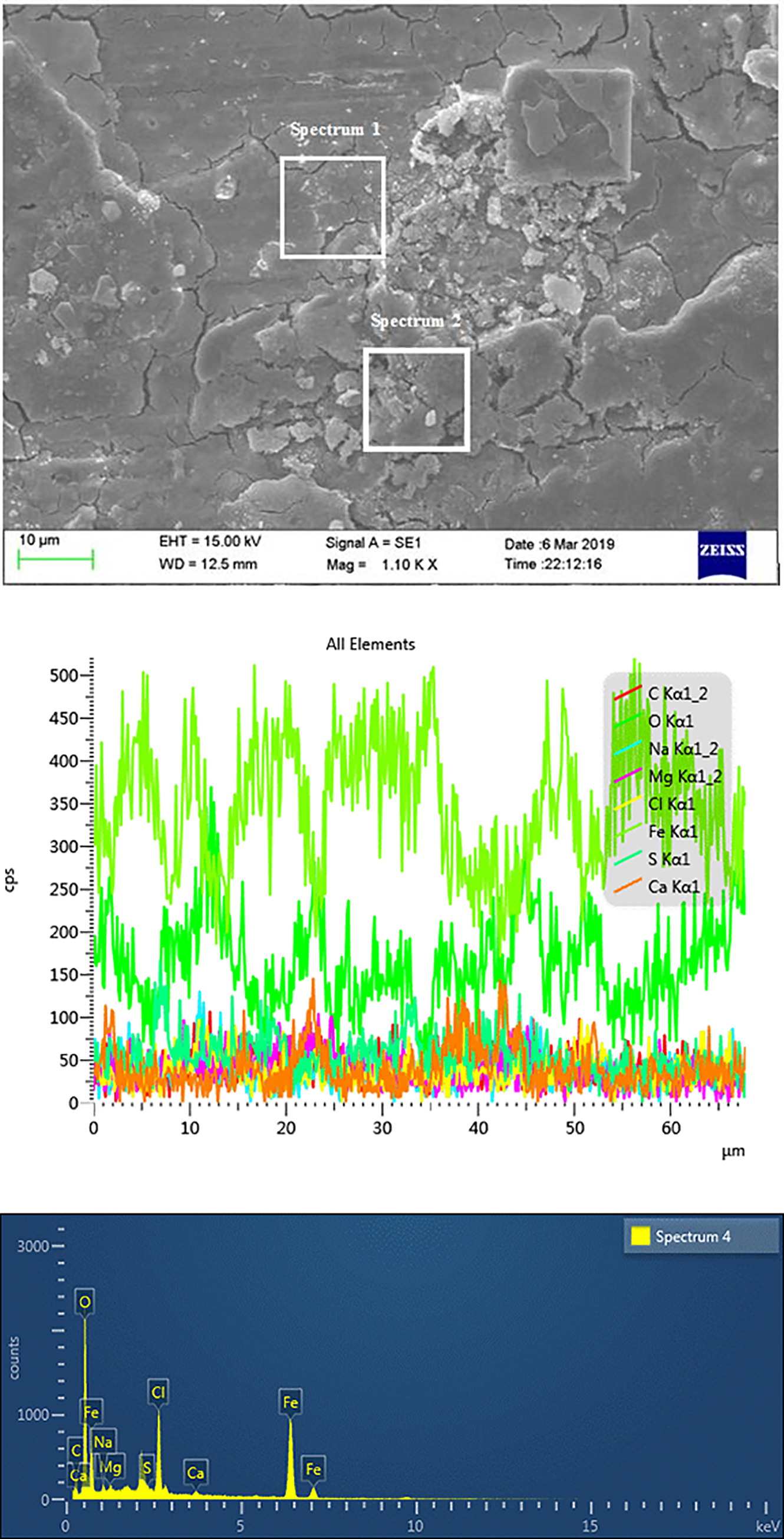

Supplement: S7 Fig — (a) Surface micro corrosion morphology, (b) Line scan of corrosion product elements on the surface and (c) Distribution of corrosion products on the surface. (TIF) [file pone.0244237.s007.tif]

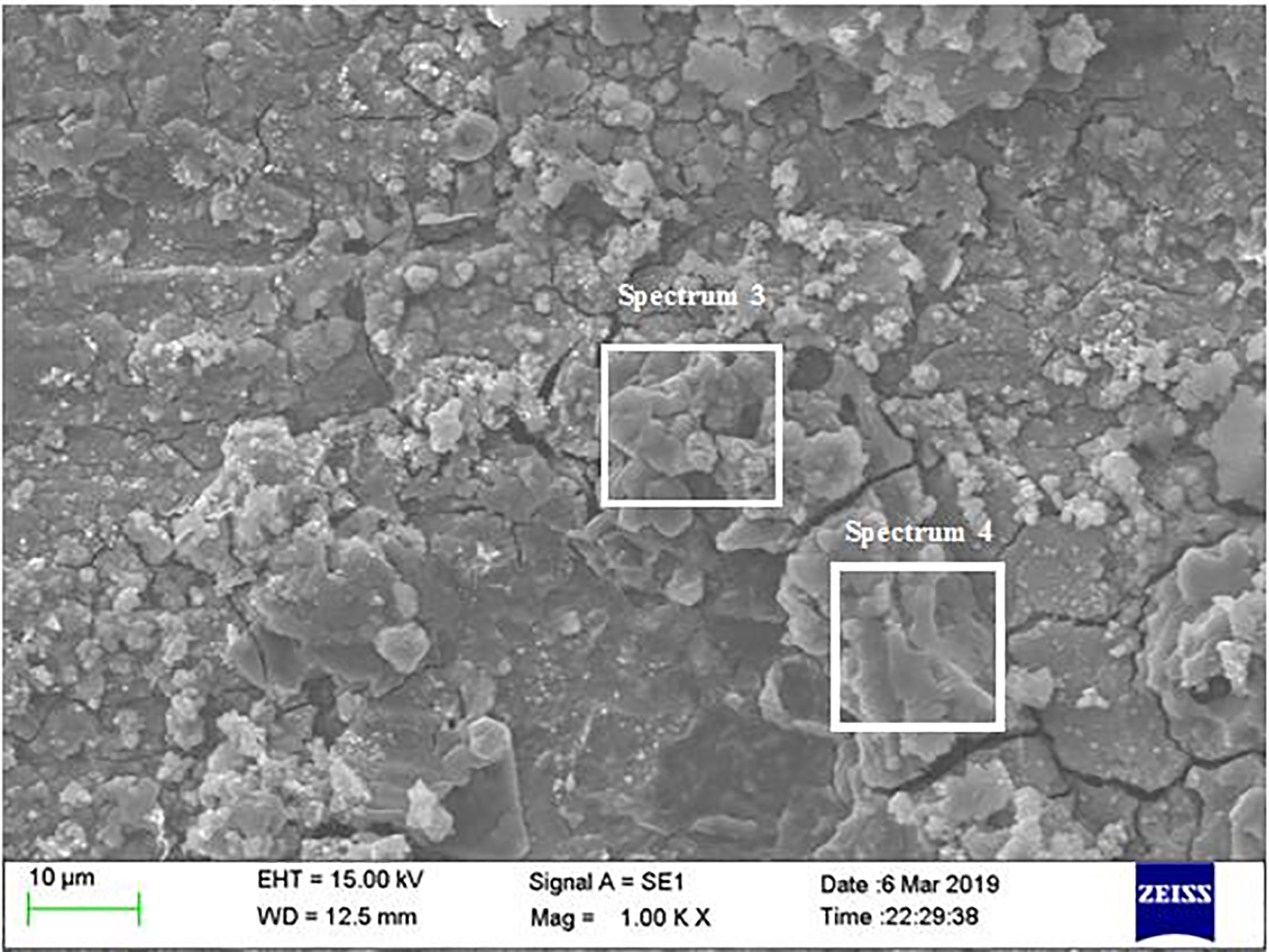

Supplement: S8 Fig — (a) Surface micro corrosion morphology, (b) Line scan of corrosion product elements on the surface and (c) Distribution of corrosion products on the surface. (TIF) [file pone.0244237.s008.tif]

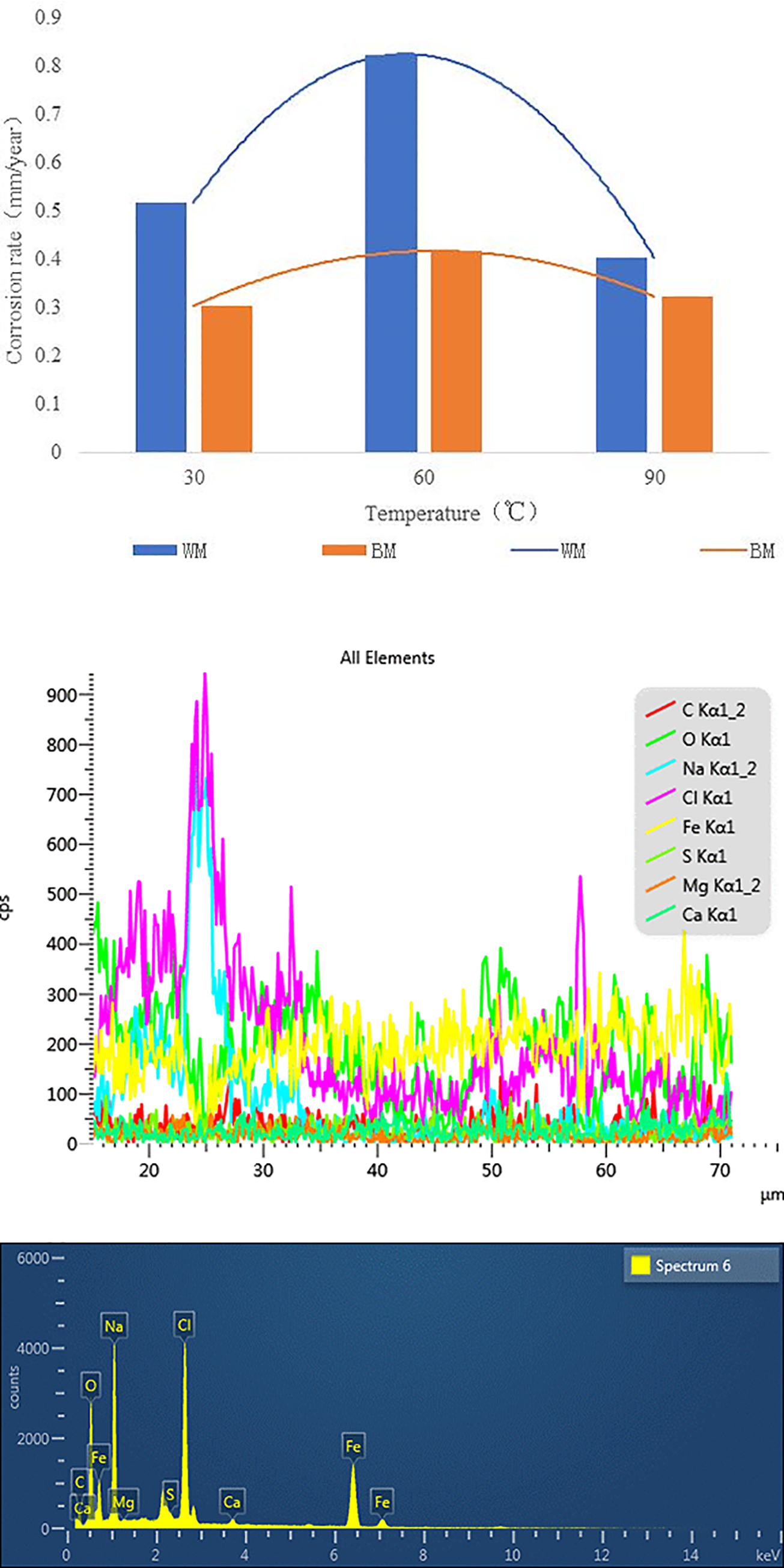

Supplement: S9 Fig — (TIF) [file pone.0244237.s009.tif]

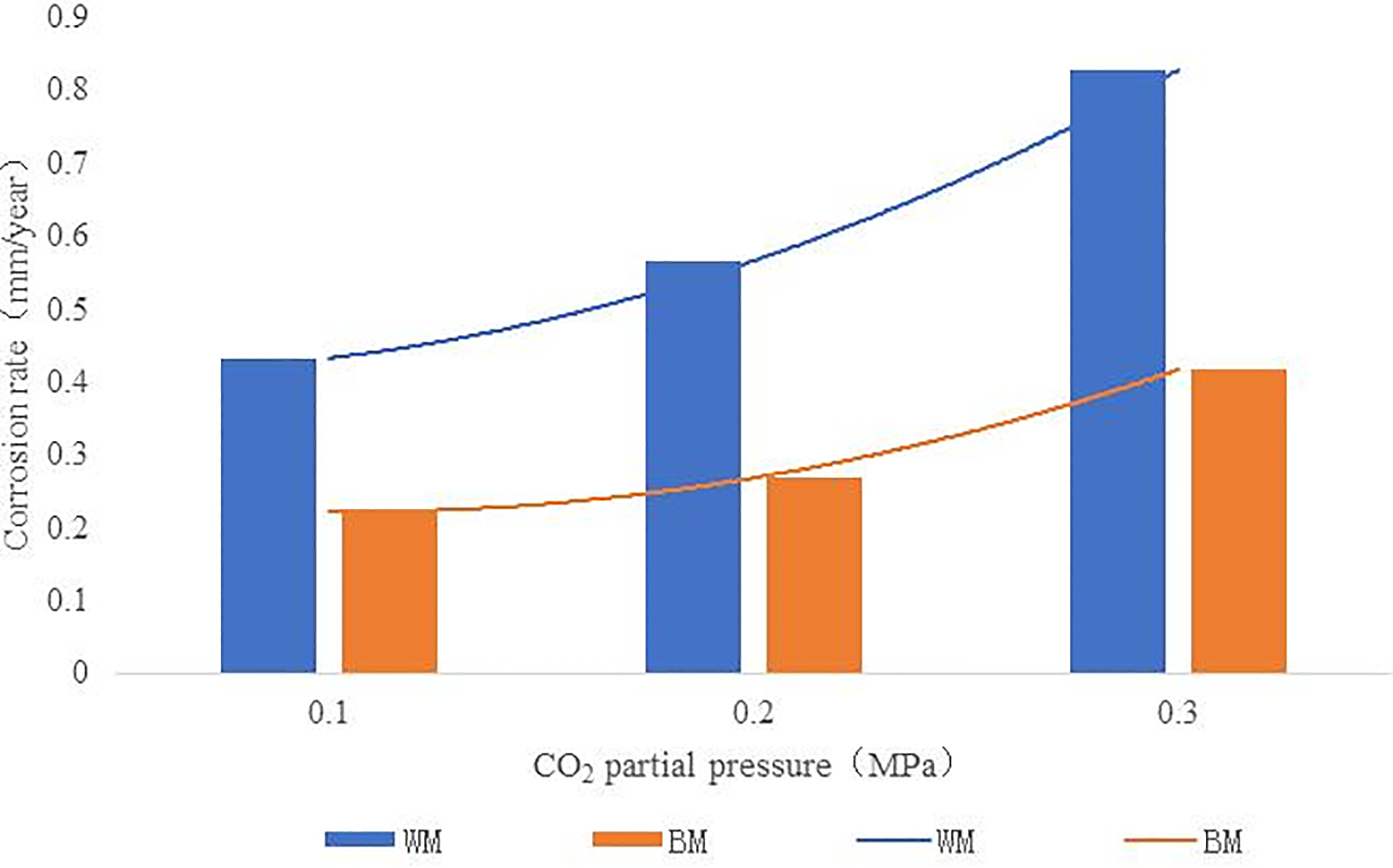

Supplement: S10 Fig — (TIF) [file pone.0244237.s010.tif]

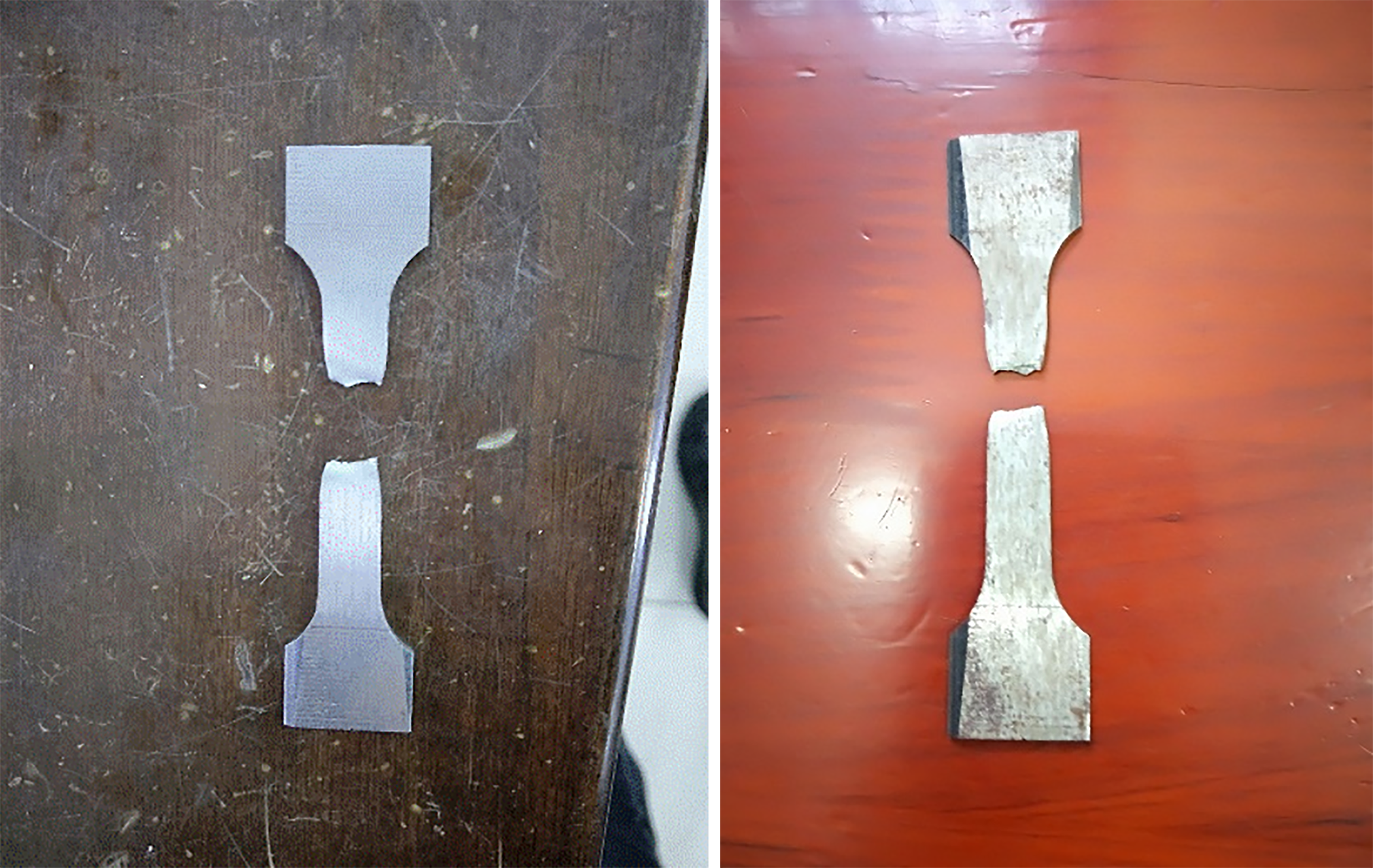

Supplement: S11 Fig — (a) Before corrosion and (b) After corrosion. (TIF) [file pone.0244237.s011.tif]

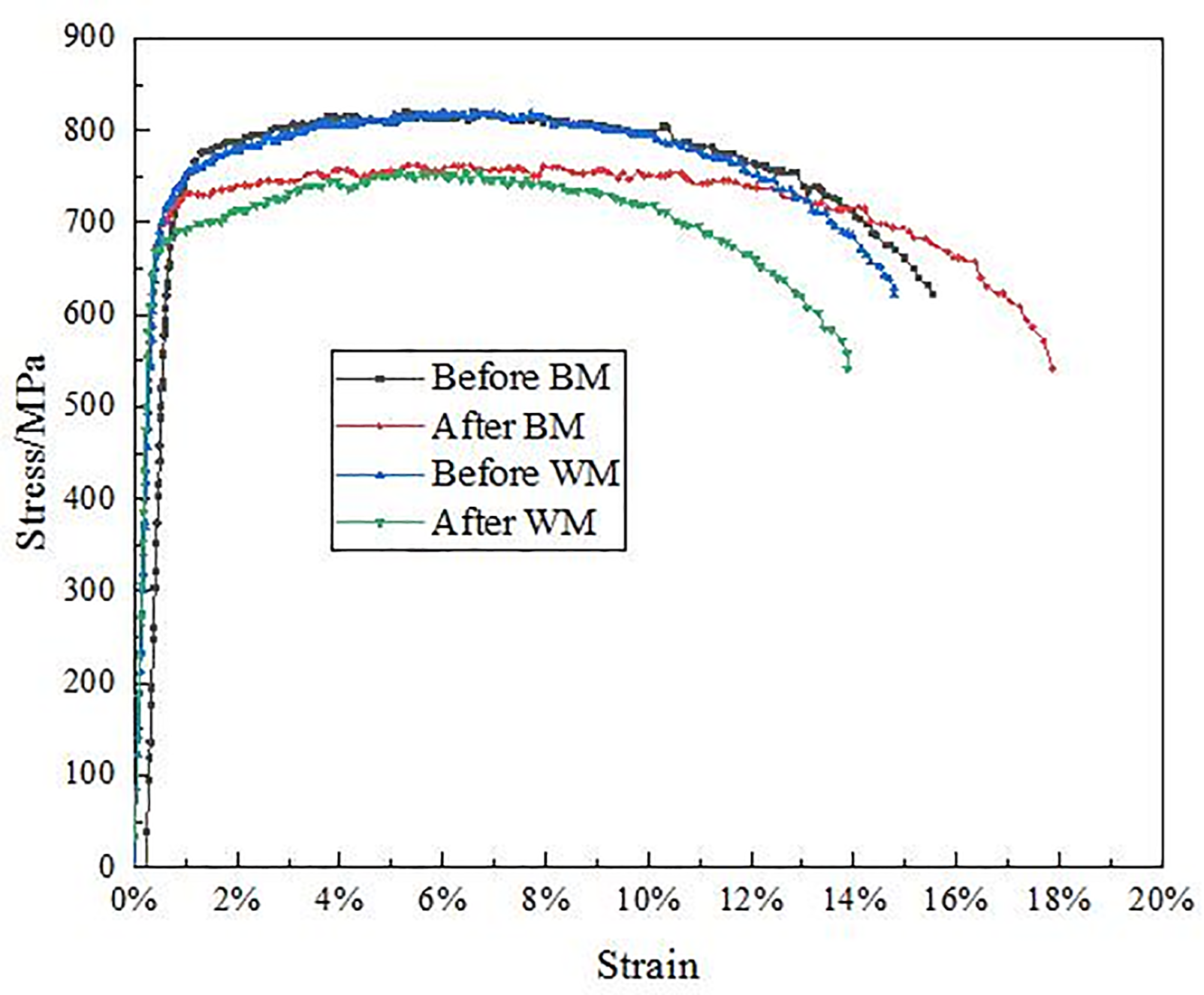

Supplement: S12 Fig — (TIF) [file pone.0244237.s012.tif]
