# Supplementary material for: Experimental study on corrosion resistance of coiled tubing welds in high temperature and pressure environment
Source: PLoS One. 2021 Jan 22;16(1):e0244237. doi: 10.1371/journal.pone.0244237 (PMC7822278; doi:10.1371/journal.pone.0244237)
Supplement: S1 Table — (DOCX) [file pone.0244237.s013.docx]

**Table 1. CT110 WM and BM chemical composition (mass fraction %).**

| **Element** | **C** | **Si** | **P** | **S** | **Cr** | **Mn** | **Fe** |
| --- | --- | --- | --- | --- | --- | --- | --- |
| BM | 0.14 | 0.39 | 0.12 | 0.05 | 0.56 | 0.48 | allowance |
| WM | 0.13 | 0.36 | — | 0.08 | 0.46 | 0.71 | allowance |
