# Supplementary material for: Experimental study on corrosion resistance of coiled tubing welds in high temperature and pressure environment
Source: PLoS One. 2021 Jan 22;16(1):e0244237. doi: 10.1371/journal.pone.0244237 (PMC7822278; doi:10.1371/journal.pone.0244237)
Supplement: S2 Table — (DOCX) [file pone.0244237.s014.docx]

**Table 2. Ion concentration of experimental reagent and drug.**

| **ion** | **Concentration(g/L)** | **drug** | **weight（g）** |
| --- | --- | --- | --- |
| $Ca^{2+}$ | 7.03 | $CaCl_{2}$ | 19.5 |
| $Mg^{2+}$ | 0.76 | $MgCl_{2}\cdot6H_{2}O$ | 6.4 |
| $SO_{4}^{2-}$ | 0.67 | $Na_{2}SO_{4}$ | 1.0 |
| $HCO_{3}^{-}$ | 0.90 | $NaHCO_{3}$ | 0.5 |
| $Cl^{-}$ | 60.0 | $NaCl$ | 74.89 |
