# Supplementary material for: Experimental study on corrosion resistance of coiled tubing welds in high temperature and pressure environment
Source: PLoS One. 2021 Jan 22;16(1):e0244237. doi: 10.1371/journal.pone.0244237 (PMC7822278; doi:10.1371/journal.pone.0244237)
Supplement: S3 Table — (DOCX) [file pone.0244237.s015.docx]

**Table 3. The analysis results of surface energy spectrum of CT BM and WM after corrosion.**

| **Element** | **C** | **O** | **Na** | **Mg** | **Cl** | **Ca** | **Fe** | **Total** |
| --- | --- | --- | --- | --- | --- | --- | --- | --- |
| BM | 9.74 | 36.32 | 1.07 | 0.61 | 11.52 | 0.72 | 39.72 | 100 |
| WM | 9.34 | 22.35 | 20.78 | 0.15 | 20.75 | 0.84 | 25.79 | 100 |
| Change/% | 4.3 | 62.5 | -94.9 | 75.4 | -44.5 | 14.3 | 54 | - |
