# Supplementary material for: Experimental study on corrosion resistance of coiled tubing welds in high temperature and pressure environment
Source: PLoS One. 2021 Jan 22;16(1):e0244237. doi: 10.1371/journal.pone.0244237 (PMC7822278; doi:10.1371/journal.pone.0244237)
Supplement: S4 Table — (DOCX) [file pone.0244237.s016.docx]

**Table 4. The comparison of mechanical properties of CT110 WM and BM before and after corrosion.**

| **sample** | **condition** | **yield strength (MPa)** | **tensile strength (MPa)** | **ratio** | **Elongation** |
| --- | --- | --- | --- | --- | --- |
| WM | before | 715.82 | 821.33 | 0.87 | 14.40% |
|  | after | 681.08 | 753.84 | 0.90 | 14.00 % |
| BM | before | 726.30 | 821.56 | 0.88 | 15.15% |
|  | after | 697.81 | 762.82 | 0.91 | 17.56% |
